# c-Met specific CAR-T cells as a targeted therapy for non-small cell lung cancer cell A549

**DOI:** 10.1080/21655979.2022.2058149

**Published:** 2022-04-04

**Authors:** Jingting Min, Chirong Long, Lu Zhang, Jiakang Duan, Honglian Fan, Fei Chu, Zhenghong Li

**Affiliations:** a Department of Basic Medical, Bengbu Medical College, Bengbu, AH, China; b Department of Life Sciences, Bengbu Medical College, Bengbu, AH, China; c Department of Hepatological Surgery, The First Affiliated Hospital of Bengbu Medical College, Bengbu, AH, China; d Department of Pharmacy, Bengbu Medical College, Bengbu, AH, China; e Department of Pharmacy, The First Affiliated Hospital of Bengbu Medical College, Bengbu, AH, China

**Keywords:** Chimeric antigen receptor, c-Met, NSCLC, adoptive cell therapy, immunotherapy

## Abstract

Non-small cell lung cancer (NSCLC) is considered to be one of the most prevalent and fatal malignancies, with a poor survival rate. Chimeric antigen receptor T cell (CAR-T) cell therapy is one of the most exciting directions in the field of Cellular immunotherapy. Therefore, CAR-T cells that target c-Met have been developed for use in NSCLC therapy and might be a potential therapeutic strategy. The anti c-Met ScFv structure was fused with the transmembrane and intracellular domains. Using a lentiviral vector to load the c-Met CAR gene, then transfected the c-Met CAR lentiviral into human T cells to obtain the second generation c-Met CAR-T expressing CARs stably. In vitro co-culture, experiments revealed that CAR-T cells have high proliferative activity and the potential to secrete cytokines (IL-2, TNF-α, and IFN-γ). c-Met CAR-T cells showed special cellular cytotoxicity in LDH release assay. A subcutaneous tumor model in nude mice was used to test the anticancer effectiveness of c-Met CAR-T cells in vivo. For c-Met positive NSCLC tissue, according to tumor volume, weight, fluorescence intensity, and immunohistochemical detection, c-Met CAR-T cells had stronger tumor growth suppression compared to untransduced T cells. HE staining revealed that c-Met CAR-T cells did not produced side effects in nude mice. Taken together, we provided useful method to generate c-Met CAR- T cells, which exhibit enhanced cytotoxicity against NSCLC cells in vitro and in vivo. Thus, providing a new therapeutic avenue for treating NSCLC clinically.

**Highlights**

(1) c-Met CAR-T capable of stably expressing c-Met CARs were constructed.
(2) c-Met CAR-T have strong anti-tumor ability and proliferation ability in vitro.
(3) c-Met CAR-T can effectively inhibit the growth of A549 cells subcutaneous xenografts.

## Introduction

Lung carcinoma is considered to be associated with the highest morbidity and mortality in the world [1], and about 75% of patients are already in the middle and advanced stages when diagnosed. Currently, the treatment regimens are mainly surgery, radiotherapy, chemotherapy, and molecular targeted drug therapy, but the 5-year survival rate is still very low [2]. Adoptive immunotherapy is widely considered to be one of the most potent cancer therapies. CAR-T-based adoptive cell immunotherapy has made a breakthrough in the treatment of hematological tumors and has been gradually applied to solid tumors [3]. CAR-T cells can enhance co-stimulatory signal transduction, overcome the inhibition of MHC and cytokine expression by tumor cells, and enhance immune responses. NSCLC CAR-T therapy is currently being investigated by researchers in the hopes of providing treatment with great potential [4,5].

c-Met is a transmembrane receptor encoded by the Met gene with autonomous phosphatase activity, belonging to the tyrosine kinase receptor superfamily, and is mainly expressed in epithelial cells [6]. Transduction of abnormal signals can promote cell proliferation, survival, tumor invasion, and angiogenesis, as well as initiate and sustain tumor transformation [7]. c-Met is a product of the proto-oncogene Met and plays a crucial role in the development of cancer [8,9]. Activated c-Met can promote cell proliferation, invasion, survival, angiogenesis, and movement. In NSCLC tissues, an elevated level of c-Met protein expression is common [10]. Multiple studies have shown that c-Met is overexpressed in 60% of cases, while phosphorylated c-Met is elevated in 40–100% of cases [11,12]. According to our previously reported studies, the humanized c-Met antibody can effectively inhibit the invasion and induce the apoptosis of NSCLC cells in vivo and in vitro. Liu [13] constructed CAR (expressing NK cells) that targets and identifies the c-Met antigen. Furthermore, the in vitro cytotoxicity tests of Liu showed that the cells could induce the lysis and death of c-Met positive targets. Based on the above studies, we selected c-Met as the target to construct CAR-T cells and explored the possibility of adopting immunotherapy for NSCLC. Since elevated c-Met overexpression levels are associated with a low survival rate in many solid tumors, such as gastrointestinal tract, head, neck, breast, ovarian, and cervical cancers. Moreover, the CAR-T cells could be used in subsequent studies to treat other types of tumors.

An elevated expression of c-Met has been found in several tumor tissues compared to healthy tissues. Past studies have shown that elevated expression of the c-Met protein in patients with NSCLC has a poor prognosis [14]. In both preclinical and clinical evaluations [15], c-Met inhibitors in NSCLC treatment showed antitumor activity. Therefore, chimeric antigen receptor therapy targeting c-Met may be a new method for the treatment of NSCLC. According to the reported studies, c-Met overexpression in tumor tissues of NSCLC patients is approximately three times greater than that in healthy tissues [16]. The c-Met elevated expression in lung cancer is correlated to the clinical stage of lung cancer [17]. Furthermore, the expression level of c-Met is correlated with the differentiation degree of lung cancer, and the lower the differentiation degree, the higher the expression level [18,19].

Based on the benefits of CAR-T in tumor treatment and the importance of c-Met in NSCLC incidence and progression. We anticipated that using c-Met as a CAR-T target may be beneficial in the treatment of NSCLC. Herein, we aimed to construct CAR-T cells targeting human c-Met protein and observe the CAR-T cells’ ability to destroy NSCLC cells expressing c-Met in vitro and in vivo. It is expected to have a powerful inhibitory impact on NSCLCand give a new method for cellular immunotherapy for NSCLC. Thus, this study provides an experimental foundation for the use of chimeric antigen receptors to treat NSCLC.

## Materials and methods

### Donor, cell line, and culture

With the approval of Bengbu Medical College’s ethical committee, blood samples were taken from healthy blood donors. Written informed consents have been obtained from all the blood donors.

The NSCLC cell line A549 was retained by our research group. Ovarian cancer cell line A2780 was donated by Dr. Qiuman Wang (Shandong University). A549-FLUC cells were obtained by infection with luciferase-carrying lentivirus GV633 and screening with puromycin.

The underlined cell lines were grown in RPMI-1640 medium (Gibcobrl, Gaithersburg, MD, USA). Media (enriched with 10% heat-inactivated FBS (Gibco) was incubated at 37°C with CO_2_ (5%).

The following reagents were purchased from Invitrogen (USA): PE-conjugated mouse anti-human CD4, PerCP/Cy5.5-conjugated mouse anti-human CD8, FITC-conjugated mouse anti-human c-Met, APC-conjugated mouse anti-human CD3 were purchased from Biolegend (USA).

### Construction of the anti c-Met chimeric antigen receptor vector and lentivirus encapsulation

The ScFv sequence (patent number: CN104159909A) of the anti c-Met antibody was used as the target gene to reassemble the linearized lentivirus vector plasmid GV401 (Shanghai Genechem Co. Ltd. China) carrying the second-generation CAR frame (the recombinant plasmid was named c-Met CAR lentivirus plasmid).

HEK293 cells were co-transfected with c-Met CAR lentivirus plasmid, plasmid VSVG, and pSPAX2 to produce lentiviral supernatant, virus suspension obtained was named c-Met CAR lentivirus. The CD19 CAR lentivirus was used as a negative control [20].

### Construction of c-Met CAR-T cells

Post dilution of peripheral blood with normal saline, continuous density gradient centrifugation, based on diluted Ficoll solution (Solarbio, Beijing, China), was performed to isolate PMBC. Anti-CD3 and anti-CD28 mAb were used to activate T cells, and 200 U/mL IL-2 was added to the media for further culturing. To perform lentivirus transduction, precoating of 48 non-tissue culture plates was carried out with 4 mg of RetroNectin in PBS (200 µL) at 4°C for 12 hours. Next, the culturing of anti-CD3/CD28 activated T cells(1 x 10^6^/mL)was conducted in the coated wells [21]. T cells were infected with c-Met CAR lentivirus and CD19 CAR lentivirus with MOI (The ratio of the virus to the number of cells at infection) of 5. Likewise, CD19 CAR-T and c-Met CAR-T were acquired. The media of CAR-T cells was changed every next day. Following that, the growth, density, and fluorescence signal of these cells were observed. The culturing of transduced cells was carried out with IL-2 for 5–10 days, followed by anti-tumor effect evaluations. The media and cytokines were changed every 2 days during culture or when passaging the T cells for splitting for expansion.

### Identification of c-Met CAR-T cells

On the 4^th^ day after lentivirus infection, the extraction of total RNA was carried out from c-Met CAR-T, CD19 CAR-T, and T cells by using Trizol [22](15,596,026, Invitrogen, USA), isopropanol, ethanol, chloroform, followed by dissolving in RNase-Free water. RNA was reverse transcribed using the PrimeScript RT reagent Kit with gDNA Eraser (RR047A, Takara, Japan), and CD3ζ was quantified using the TB Green Premix Ex Taq (RR820A, Takara, Japan) [23], as suggested by the manufacturer’s instructions. GAPDH was used as internal control and calculated by the 2^−ΔΔCt^ method. The primer sequences are shown as follows: CD3ζ:(forward) 5′- CGGAGGCCTACAGTGAGATT-3′, (reverse) 5′-AGACCCTGGTAAAGGCCATC -3′; GAPDH: (forward) 5′- GAAGGTGAAGGTCGGAGTC-3′, (reverse) 5′- GAAGATGGTGATGGGATTTC-3′.

Furthermore, c-Met CAR-T, untransduced T cells, and CD19 CAR-T were harvested and lysed by RIPA Lysis Buffer [24,25] (R0020, Solarbio, China). The BCA protein analysis kit (2,161,296, Thermo Scientific, Waltham, MA) was used to evaluate the protein concentration. SDS polyacrylamide gel electrophoresis was carried out to separate the protein samples, followed by transferring on to the PVDF membrane through the solid carrier. The membrane incubation was then carried out with primary antibody including mouse-anti-human CD3ζantibody (Lot#B9421, Santa Cruz, USA)/GAPDH Polyclonal antibody (BL006A, Biosharp, China), followed by incubation with secondary antibody (conjugated with HRP) *i.e*., Goat-Anti- mouse IgG (BL001A, Biosharp, China). Moreover, the DAB chromogenic method was used to evaluate the exogenous CD3ζ of CAR structure fusion protein.

### Flow cytometry

A BD FACS Celesta flow cytometer was employed to conduct flow cytometry. FlowJo 10.4 was used to analyze the flow cytometric data. Next, PBS was utilized to rinse (thrice) the cells, followed by incubating the cells with and then incubating with antibodies for 60 min at 4°C in the dark. The underlined cells were rinsed (thrice), followed by resuspending in PBS (200 ml) before evaluation.

Infection efficiency: On day 6, an EGFP positive rate was identified. The positive rate of CAR-T was measured by the proportion of green fluorescence detected in the FITC channel of the flow cytometer [26]. c-Met CAR-T cells (5 × 10^5^) were collected and washed (thrice) with PBS. APC anti-human CD3(Cat3173018, Biolegend, USA), PE anti-human CD4 antibody (12–0049-42, Invitrogen, USA), and Percy5.5 anti-human CD8 antibody(45–0088-42, invirtrogen, USA) were stained in dark at 4°C. After washing three times with PBS, CD4^+^ and CD8^+^ T cells were quantitatively evaluated by flow cytometry [27], and the obtained results were analyzed by Flowjo.

The A549 cells were digested with 0.25% trypsin, and a FITC-labeled anti-human c-Met antibody (11–8858-42, Invirtrogen, USA)was added to stain these cells. The cells were incubated at 4°C for 1 h. After repeated washing, the cells (5 × 10^5^) was resuspended a 200 µL buffer [28].

### Immunohistochemistry

From January to December 2019, 40 cases of NSCLC tumor tissues were gathered from tumor patients at Bengbu Medical College’s First Affiliated Hospital’s Department of Thoracic Surgery. As controls, another 20 cases of paracancerous lung tissues from lobectomy specimens were chosen. NSCLC and paracancerous lung tissue samples were fixed in 4% neutral formalin, embedded in paraffin, cut into 4 μm thick serial sections, and dried. The antibody was diluted at 1:2000 (ab216574, Abcam, USA) and applied to slides for 16 h at 4 ^o^C. Goat anti-rabbit secondary antibody was incubated for 15 min. Immunohistochemistry findings were blindedly assessed by two competent diagnostic physicians in the pathology department. Evaluation and analysis were performed according to the positive rate of cells and staining intensity, and the positive rate was scored as 0 points (0%), 1 point (0 ~ 10%), 2 points (10%~50%), 3 points (> 50%), staining Intensity score: 0 (no tinting), 1 (light tinting), 2 (medium tinting), 3 (dark tinting). The positive ratio score × staining degree score of each section is the final score. 0 to 1 was divided into -, 2 was divided into ±, 3 ~ 5 were divided into +, 6 ~ 8 were divided into ++, > 8 were divided into +++.

### Antigen-specific blastogenesis assay

**P**ost in-vitro culturing of healthy donor effector T cells (c-Met CAR-expressed, CD19 CAR-expressed or non-transduced T cells) for 10 days, effector T cells were co-cultured with c-Met positive and negative cell lines (A549 and A2780 in a 5:1 effector target ratio), and then CCK-8 reagents (BS350A, Biosharp, China) were added at 24, 48, and 72 h [29], respectively. The absorbance of cell supernatant was recorded at 450 nm after 2 hours of culture at 37°C.

### Cytotoxicity assays

LDH based assay was conducted to evaluate the cytotoxic effects of CAR-T cells [30]. Tumor cells (1 x 10^5^ cells /well) were added to the 96-well plates, and then according to the effect of target ratio 1:1, 5:1, and 20:1, c-Met CAR-T, CD19 CAR-T, and non-transduced T cells were added. Next, the incubation was performed at 37°C and CO_2_ (5%) post 6 hours of culture.And LDH detection reagent was added (C0017, Beyotime, China), the optical density was recorded at 490 nm wavelength.

The mean value of the three wells was substituted into the following formula to calculate the killing efficiency % = (the measured hole – effector cells natural release OD value – target cells natural release OD value + blank control OD value)/(the maximum release hole OD value of the target cells – target cells natural release OD value).

### Cytokine release assays

The overnight co-culturing of c-Met CAR-T, CD19 CAR-T, and untransduced T cells was carried out with cancer cells (1 × 10^5^ cancer cells/well) in a 5:1 ratio in a 96-well plate in 200 µL media, followed by collecting the supernatant of the cells. Next, the secretion of IFN-γ, TNF-α, and IL-2 by the effector cells was determined using the ELISA kit [31](ml077386/ml077385/ml058063, Mlbio, China).

### In vivo experiments

BALB /c nude mice aged 5 to 6 weeks were selected as experimental animals, purchased from Changzhou Cavens Laboratory Animal, and reared in a special barrier system for routine monitoring.

On day 0, A549-Fluc cells (3.0 × 10^6^ cells) dissolved in PBS (100 μL) were injected subcutaneously into the flanks of nude mice. The size of the tumor and body weight were analyzed every 3 days (tumor size = 1/2× long diameter × short diameter ^2^) [25]. Tumor-bearing nude mice were randomly divided into three groups: activated T cell group, and PBS group (n = 5 each) after the mean tumor size of 100 mm^2^ on the 14th day of inoculation. On the 14th day, the CAR-T cell group was injected with 1.0 × 10^7^ CAR-T cells. This procedure was repeated one week later, and nude mice in the control group were exposed to PBS or equivalent amounts of untransduced T cells.

On the 28th day, the mice were euthanized by cervical dislocation, followed by removal of the liver, spleen, lung, and kidney tissues of the nude mice, and then overnight fixing was carried out by using paraformaldehyde (4%). After that, paraffin-embedded sections were performed, and H&E staining [32] was performed. In vitro, the tumor tissues were apoptosis-associated Tunel staining and immunohistochemically stained with proliferation-associated antigen Ki67 to evaluate the effect of c-Met CAR-T cells on the progression of established xenograft tumors [25]. Immunostaining of tumor tissue sections (frozen) with anti c-Met antibodies (37–010), Invitrogen, USA) was carried out to evaluate the expression of target antigens in tumor tissues after treatment with c-Met CAR-T cells.

### Statistical evaluations

The obtained data were indicated as mean ± SD (x ± s),, and enumeration data as a percentage (%). SPSS software (version 23.0) was employed for statistical evaluations. The one-way ANOVA was carried out to compare the two groups.

## Result

We investigated whether c-Met CAR-T cells can be effectively generated and had a high killing potential in vitro and in vivo against the non-small cell lung cancer cell line A549. We expected c-Met CAR-T cells to have improved activation and anti-tumor efficacy. As a result, the purpose of this study was to see whether c-Met CAR-T successfully suppressed A549 cells growth and promoted apoptosis, and to see whether CAR-T therapy targeting c-Met might be used to treat NSCLC.

### Designing and identification of the c-Met CAR lentivirus expression plasmid

**Figure 1.**
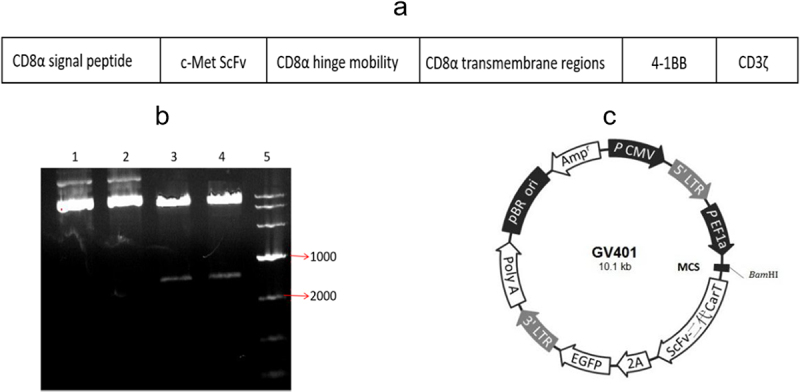
CA*R plasmid* construction and validation. (a) c-Met CAR structure diagram; (b) Agarose gel electrophoresis of plasmid digestion; (c) Schematic diagram of lentiviral vecto*r plasmid* structure.

The gene fragment of c-Met CAR was composed of CD8α signal peptide, c-Met ScFv, CD8α hinged region, CD8α transmembrane region, 4–1BB, and CD3ζ, as indicated in Figure 1(a). Sequencing confirmed the successful construction of the clone.

After the gene fragment of c-Met CAR was inserted into the lentiviral vector plasmid, the recombinant plasmid was digested by BamHI/BamHI enzyme. Following that, 1% AGAR gel electrophoresis was employed to identify the digested product, as depicted in Figure 1(b). The obtained data indicated successful construction of the plasmid. Moreover, two bands appeared in the digested plasmid (lane 3 and lane 4), which matched the size of the vector plasmid and the target gene, while the undigested plasmid (lane 1 and lane 2) had no corresponding bands of the target gene.

Penicillin resistance genes in recombinant plasmids can be used to select suitable *Escherichia coli* colonies. And the ScFv second-generation CAR-T sequence and the marker gene EGFP are expressed by the same promoter (Figure 1(c)), and the cells carrying green fluorescence can be considered as the judgment the basis for the success of lentivirus infection.

### Production of c-Met targeted effector T cells

**Figure 2.**
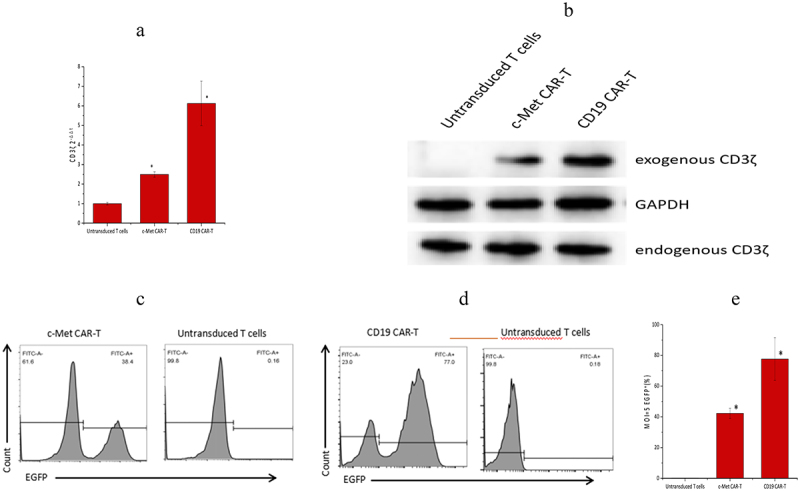
Validation of gene and protein levels of second-generation CAR molecules, (a) PCR detection for the presence of second-generation CAR sequences in c-Met CAR-T cells (*p ≤ 0.01 vs* Untransduced T cells) Note: Lane 1 is genomic DNA of c-Met CAR T cells, lane 2 is genomic DNA of activated T cells, and lane 3 is reference TAKara DNA marker. (b) Western blot to detect CD3ζ expression in c-Met CAR-T cells. Exogenous CD3ζ shows the molecular weight of the fusion protein, and the molecular weights of CD19 CARs and c-Met CARs are both around 55KD. (c) Infection efficiency of c-Met CAR-T lentivirus, expressed EGFP. Gating strategy used to determine EGFP surface expression on c-Met CAR-T was established based on blank control (Untransduced T cells), FITC mode with the same wavelength as EGFP was used for detection .Numbers represent the percentage of gated cells. (d)Infection efficiency of CD19 CAR-T lentivirus, expressed EGFP. Gating strategy used to determine EGFP surface expression on CD19 CAR-T was established based on blank control (Untransduced T cells), FITC mode with the same wavelength as EGFP was used for detection. Numbers represent the percentage of gated cells. (e)The statistical analysis of lentivirus infection efficiency.

c-Met CAR-T and CD19 CAR-T were successfully prepared by lentivirus infection. Next, qRT-PCR was performed, which revealed that, relative to the untransduced T cells, the CD3ζ mRNA level of c-Met CAR-T and CD19 CAR-T was found to be considerably enhanced post-infection at lentivirus (*p* *< 0.01*), as shown in Figure 2(a).

Western blotting results revealed that c-Met CAR-T cells and CD19 CAR-T cells express both exogenous and endogenous CD3ζ, whereas untransduced T cells solely express endogenous CD3ζ, as indicated in Figure 2(b).

Furthermore, an increased infection efficiency was observed across c-Met CAR and CD19 CAR lentivirus (at MOI = 5). According to the obtained data, the infection efficiency of c-Met CAR-T and CD19 CAR-T was 41.90 ± 3.22%, and 77.67 ± 13.91%, accordingly (Figure 2(c~e)).

### Characterization of c-Met CAR-T cells and tumor cells

**Figure 3.**
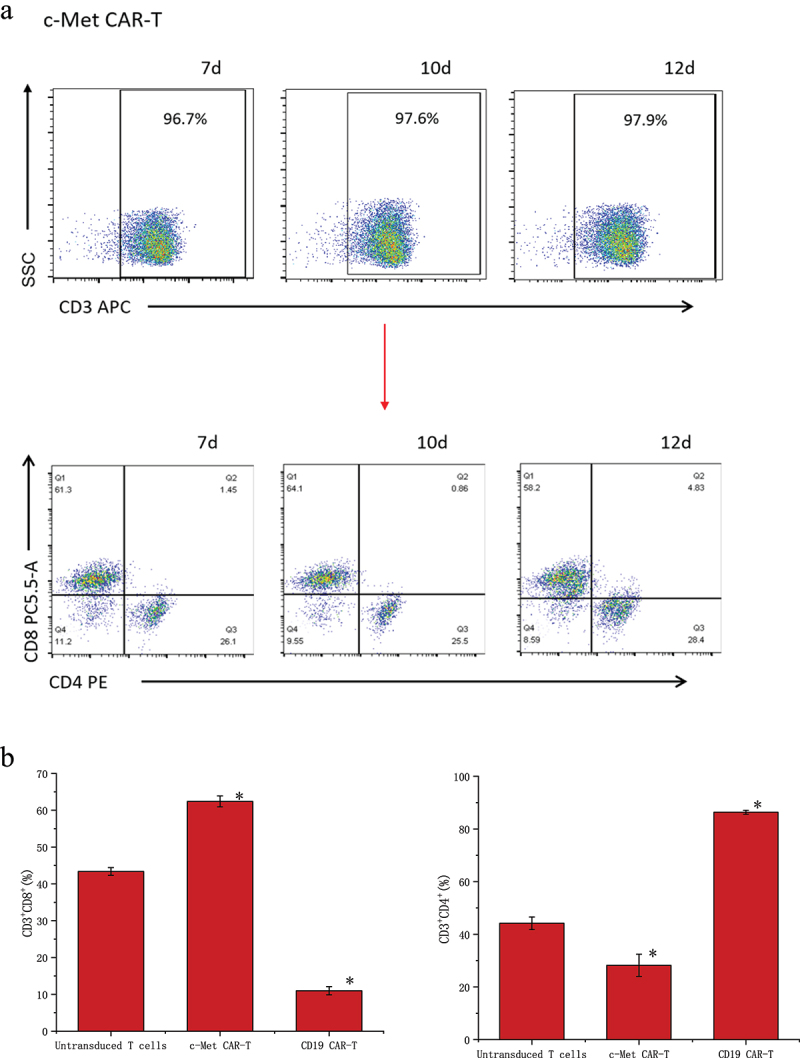
Continued.

**Figure 4.**
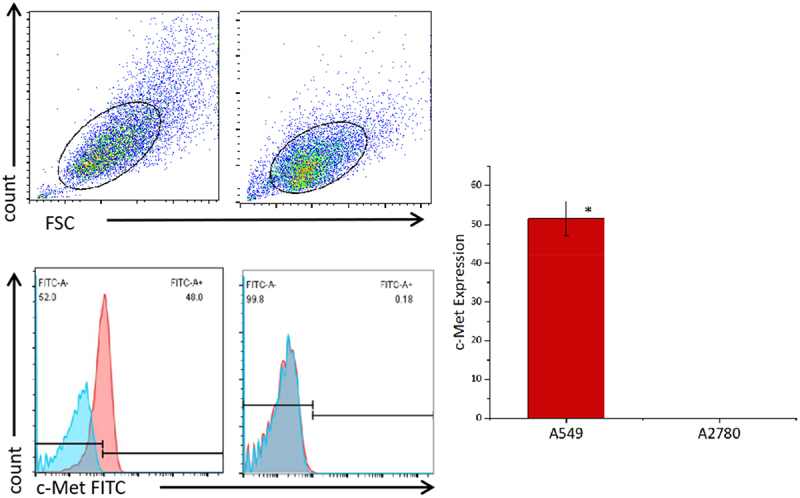
Expression levels of c-Met on the surface of cancer cell lines detected by flow cytometry. A549 cells and A2780 cells were stained with FITC conjugated anti c-Met mAb(red),Unstained cells were used as blank control(blue).The results represent the mean of 3 independent experiments. Histogram data are represented as mean ± standard deviation (N = 3) of relative FITC expression levels as compared to blank control.Two sample paired Student’s t-tests were used to determine statistical significance. (*p < 0.01 vs the c-Met expression of A2780).

The data detected by flow cytometry was gated by CD3^+^ cells to analyze the proportion of CD3^+^CD4^+^ and CD3^+^CD8^+^ cells (Figure 3(a)). The results showed that CD4^+^ and CD8^+^ accounted for 44.2 ± 2.36% and 43.37 ± 1.06% of untransduced T cells. CD4^+^ and CD8^+^ accounted for 28.23 ± 4.23% and 62.4 ± 1.50% of c-Met CAR-T cells. CD4^+^ and CD8^+^ accounted for 86.33 ± 0.72% and 10.99± ± 1.14% of CD19 CAR-T cells (Figure 3(b)). The proportion of CD8^+^T cells and CD4^+^T cells was found to be up-regulated post lentiviral infection in c-Met CAR-T and CD19 CAR-T, accordingly. Flow cytometric analysis revealed that c-Met expression was  52.6± 4.22% in NSCLC cells A549, but was not detected in non-target A2780 cells, as shown in Figure 4.
**Figure 3.**
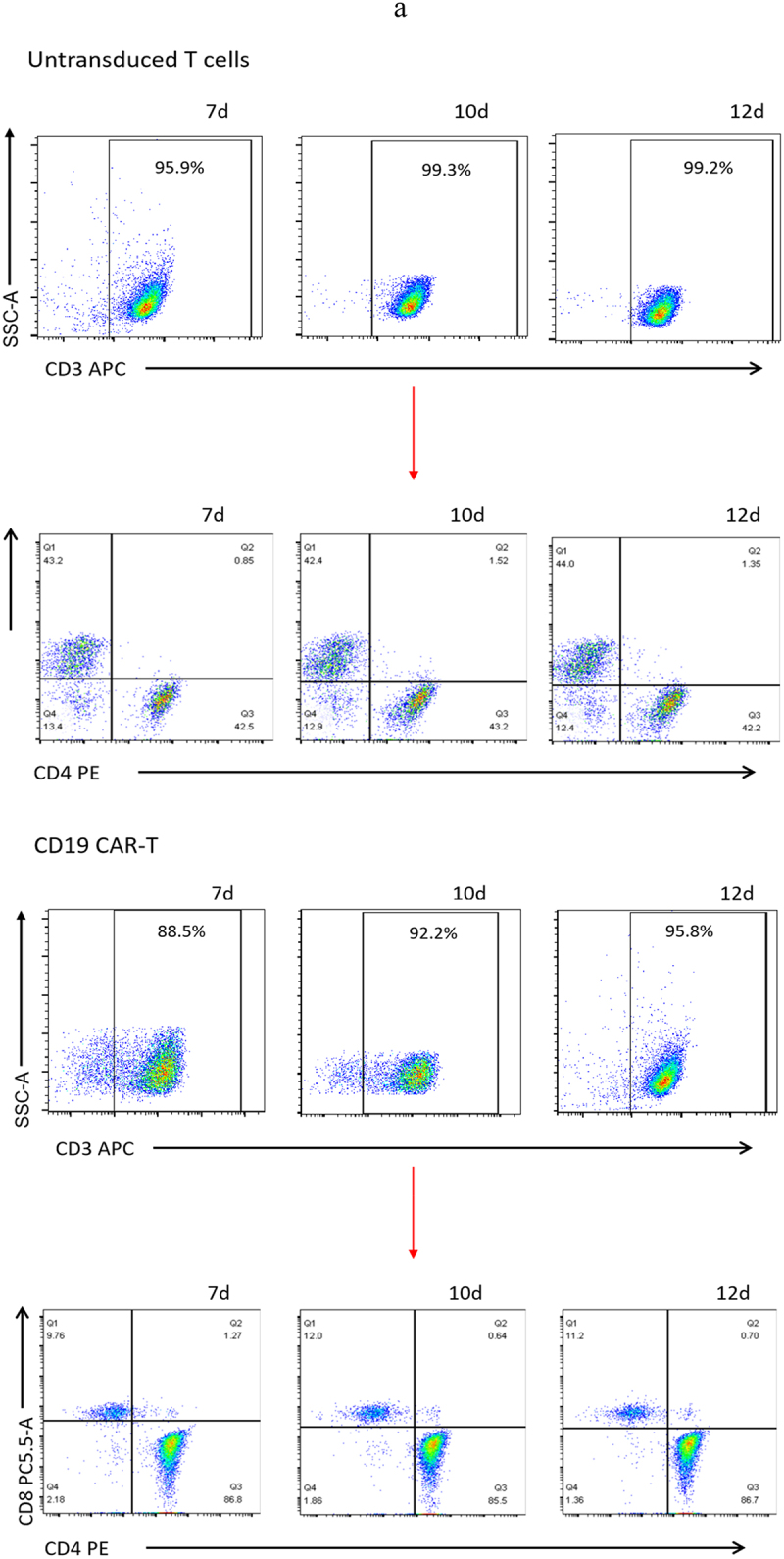
Proportion of T cell subtypes before and after lentivirus infection. (a) Flow cytometric analysis of c-Met CAR, CD19 CAR and CD4, CD8 subtypes of activated T cells; CD4^+^ and CD8^+^ proportion in c-Met CAR-T, CD19 CAR-T and Untransduced T cells are shown. From day 7 to day 12. The cells were stained with PE conjugated anti-CD4 mAb and PC-5.5 conjugated anti-CD8 mAb, followed by fixed with paraformaldehyde, and analyzed flow cytometry.The numbers indicated in each panel indicate the proportion of cells in the designated area. (b) Statistical analysis of c-Met CAR-T, CD19 CAR-T and CD4^+^, CD8^+^ subtypes of c-Met CAR-T, CD19 CAR-T and activated T cells. Summary of the results from the 3 individual experiments described the mean ± SEM, Two sample paired Student’s t-tests were used to determine statistical significance.(*p < 0.01 vs Untransduced T cells).

### The ability of T cells to expand in the presence of antigen

**Figure 5.**
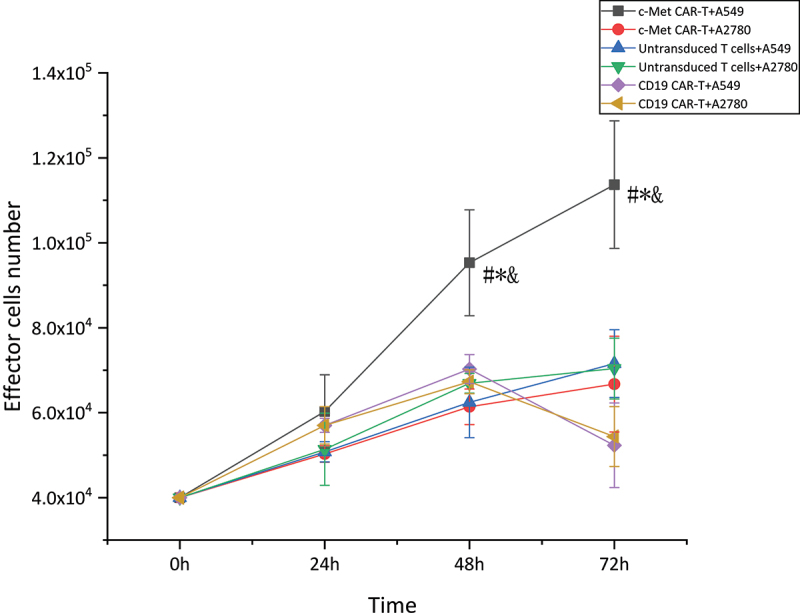
Proliferation activity of CAR-T cells stimulated by target antigen,CCK8 assay for enumeration of c-Met CAR-T which were stimulated by A549 cells.4 × 10^4^ of c- Met CAR-T, D19 CAR-T or untransduced T cells were incubated with 8 × 10^3^ A549 cells in 24 h, 48 h, 72 h. Add ccK-8 reagent and reaction at 37°C for 2 h before assay was developed,The number of cells was calculated according to the OD value.The results represent the mean of 3 independent experiments Line chart showing the represented as mean ± standard deviation of overall number of c-Met CAR-T,CD19 CAR-T or untransduced T cells.The data display the cells number change with time. Data of experiments conducted in triplicate are analyzed by two sample paired Student’s t-test comparing A549 cells stimulated c-Met CAR-T with A2780 stimulated c-Met CAR-T and comparing A549 cells stimulated c-Met CAR-T with A549 stimulated CD19 CAR-T or untransduced T cells(set to 1).*p* < 0.05 are considered significant (*p < 0.05 vs CD19 CAR-T+ A549;^#^*p < 0.05 vs* T+ A549;^&^*p < 0.05 vs* c-Met CAR-T+ A2780).

To assess the activation of CAR-T cells across target cells, parallel co-cultures were set up with A549 cells expressing c-Met and non-target cells A2780 cultured as adherent cells, were co-cultured with effector T cells in parallel co-cultures (at a 5:1 E: T ratio). Effector T cell numbers were detected at 24 h, 48 h, and 72 h. In contrast to the CD19 CAR-T and untransduced T cells, the proliferative potential of c-Met CAR-T cells was considerably enhanced in the presence of the target antigen at 48 h and 72 h. However, in the presence of non-target antigen, considerable variations were not observed in the proliferative potential of c-Met CAR-T cells relative to the CD19 CAR-T and untransduced T cells. While the activation of CD19 CAR-T and untransduced T cells A549 and A2780 was not considerably varied, as shown in Figure 5 (*p < 0.01).

### Expression of c-Met in the para-carcinoma tissues as well as NSCLC tissues

**Figure 6.**
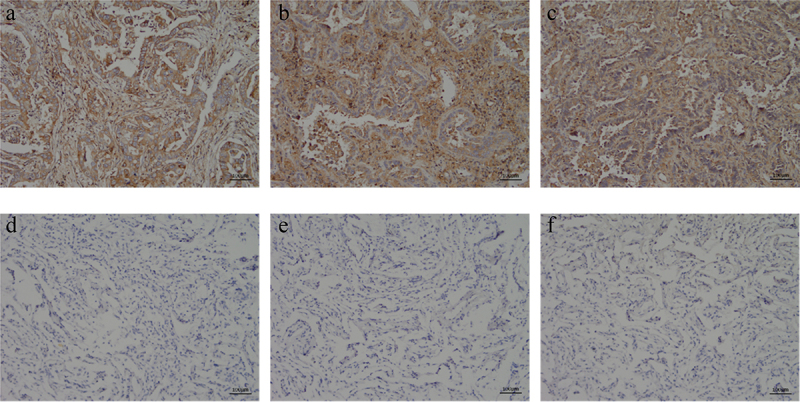
Immunohistochemical detection of c-Met expression in NSCLC and its adjacent adjacent tissues. (a) NSCLC clinical specimens IHC with moderate (IHC++), (b and c) NSCLC clinical specimens IHC with strong (IHC+++) staining, respectively. (D ~ F) The paracancerous tissue specimen was negative for c-Met based on IHC analysis(×400).

The immunohistochemical results were blindly evaluated by two professional diagnosing physicians in the pathology department. c-Met positivity was determined by the presence of brown-yellow granules in the cytoplasm and/or cell membrane. The percentage of positive c-Met expression was 15% (3/20) in 20 cases of adjacent lung tissues and 100% (40/40) in 40 cases of NSCLC tissues. Strong positive (+++) accounted for 80% (34/40), and there was a significant difference in the positive rate of c-Met expression between the two groups (p < 0.05) (Table 1, Figure 6)

**Table 1.** Expression of c-Met in NSCLC and para-carcinoma tissue

Item		c-Met					
		c-Met-	c-Met±	c-Met+	c-Met++	c-Met+++	Total
IHC	NSCLC tissue	0	0	1	6	34	40
	para-carcinoma tissue	17	3	0	0	0	20

### In vitro toxicity of c-Met CAR-T cells in NSCLC

**Figure 7.**
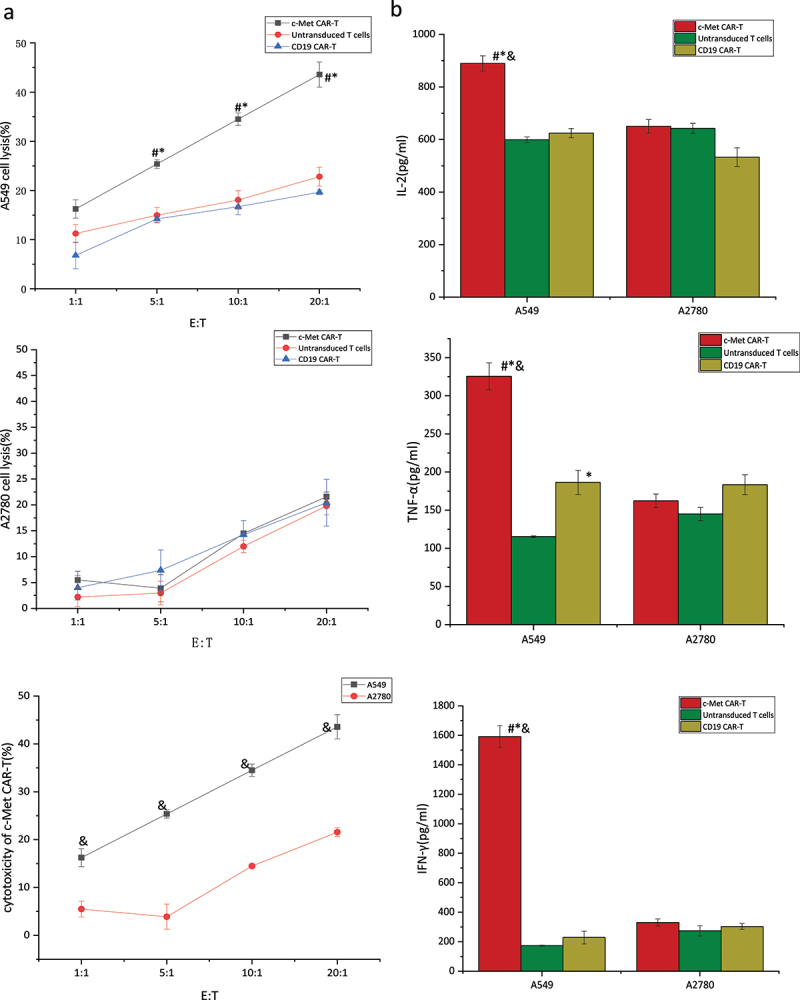
Targeted killing of c-Met CAR T cell.c-Met CAR-T or CD19 CAR-T or untransduced T cells cocultured with A549 cells at E:T = 1:1, 5:1, 10:1 or 20:1 for 6 hours. (a) Cytotoxicity of c-Met CAR-T cells against A549 or A2780 cells was determined by LDH assay. The results represent the mean of six independent experiments. Line graph data reflect the mean ± SEM of experiments conducted in triplicate are anlysis by two sample paired Student’s t-test comparing cytotoxicity of c-Met CAR-T to A549 cells with cytotoxicity of CAR-T to A2780 cells and comparing cytotoxicity of c-Met CAR-T to A549 cells, with cytotoxicity of CD19 CAR-T or untransduced T to A549 cells cells.*p < 0.05* are considered significant. (b)Cytokine release was determined by ELISA assay.Bar graph data reflect the mean ± SEM of experiments conducted in triplicate are anlysis by two sample paired Student’s t test comparing cytokine release of A549 cells stimulated c-Met CAR-T with A2780 stimulated c-Met CAR-T and comparing cytokine release of A549 cells stimulated c-Met CAR-T with A549 stimulated CD19 CAR-T or untransduced T cells(set to 1).p < 0.05 are considered significant (*P < 0.05 vs CD19 CAR-T+ A549;*^#^P < 0.05 vs* T+ A549;*^&^P < 0.05 vs* c-Met CAR-T+ A2780).

Next, the specificity and effectiveness of the c-Met CAR against c-Met-expressing targets were studied. The CAR- T cell targets were A549 cells expressing c-Met, with non-modified A2780 cells serving as a non-target control. The tumor cells were co-cultured with c-Met CAR-T, untransduced T cells, and CD19 CAR-T (Figure 7(a)). The LDH release assay was carried out to evaluate the target cells’ viability. The cell lysis rate was low in the CD19 CAR-T group, untransduced T cell group, and non-target group, and did not increase with the target cell ratio. c-Met CAR-T cells, on the other hand, destroyed c-Met^+^A549 cells effectively, with a lysis rate that was substantially greater than the other groups and increased with the target cells ratio. After co-culturing for 6 hours at a 20:1 target cell ratio, the target cell lysis rate of c-Met CAR-T cells with target cells was 43.58%.

αγ incubated with A549 or A2780 cells and cytokines including TNF-α, IL-2, and IFN-γ. Next, an ELISA was performed to detect cytokines in the supernatant post-24 hours’ co-culture. Figure 7(b) indicated the cytokine secretion in c-Met CAR-T, CD19 CAR-T group, and untransduced T cell group in the presence of target and non-target cells Relative to the CD19 CAR-T control group and untransduced T cells control group, the secretion of cytokines including IL −2, TNF-α, and IFN-γ was considerably elevated in c-Met CAR-T cells at the 5:1 of effector to target ratio. Furthermore, the secretion of cytokines (TNF-α, and IFN-γ) was elevated in c-Met CAR-T cells in the presence of target cells, as compared to the non-target group. This could be because the IL-2 added to T cell culture cannot be completely eradicated during the co-culture procedure, as seen by the presence of elevated IL-2 levels in the CD19 CAR-T control group, the untransduced T cell control group, and the non-target group. However, IL-2 levels in co-cultured c-Met CAR-T cells with target cells remained significantly greater than those in the preceding groups.

### Effect of c-Met CAR-T cells on NSCLC xenograft in nude mice

**Figure 8.**
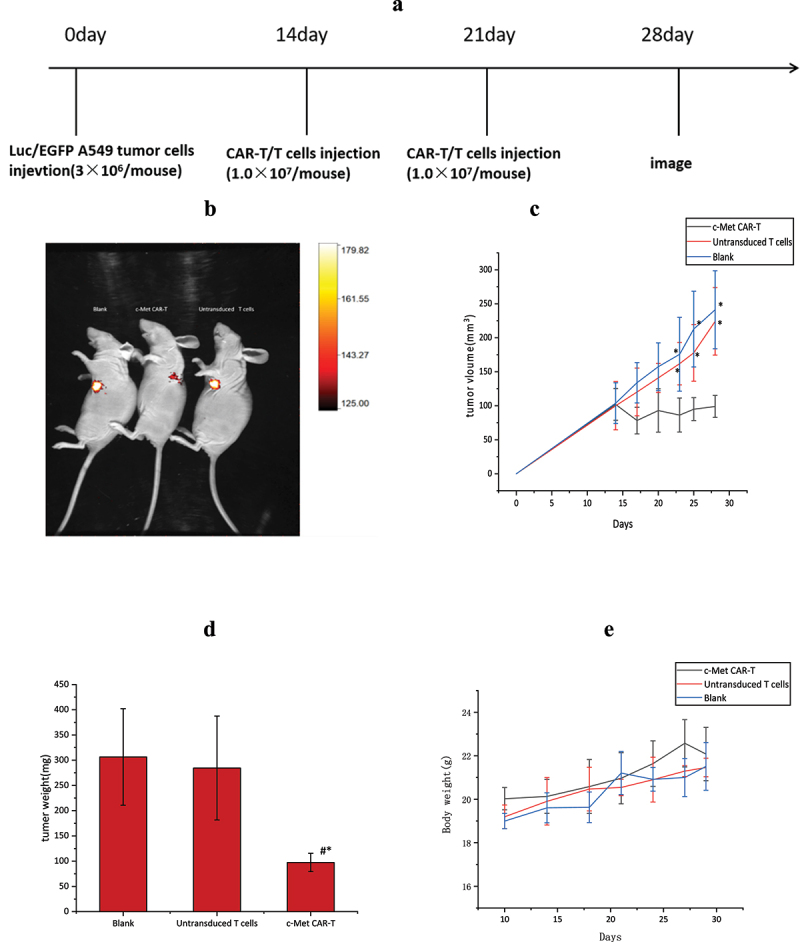
Anti-tumor effects of the anti c-Met CAR-T cells in subcutaneously implanted tumor model in nude model of human NSCLC. (a)A549-Luc (3 × 10^6^ cells) with Matrigel were injected Subcutaneous tissue below right foreleg into the space of nude mice on day 0, followed by iv injection of 1 × 10^7^ CAR-T or Untransduced T cells or same volume of PBS on day 14 and 17,. Tumor growth was assessed using vernier caliper; (b) Representative bioluminescence images of the mice are shown.; (c): line graph displays Size of tumor increased with time is shown as mean ± SEM. Data from 5 independent experiments are combined (N = 5*P < 0.05 vs CAR-T group); (d)Bar graph displays Tumor quality of nude mice in each group after treatment is shown as mean ± SEM. Data from 5 independent experiments are combined (N = 5,*p < 0.05 vs Untransduced T cells group, *p < 0.05 vs* Blank group).;(e)Line graph displays body weight fluctuation curve of nude mice in each group during treatment is shown as mean ± SEM. Data from 5 independent experiments are combined (N = 5).

14 days after subcutaneous infusion of A549-fLuc cells in nude mice, the tumor volume averaged 100 mm^3^. Next, the impact of c-Met CAR-T cells on NSCLC was evaluated (Figure 8(a)). Tumor bioluminescence imaging (Figure 8(b)) and ververine caliper, and electronic scales measurements showed that c-Met CAR-T cells considerably attenuated the growth of tumor cells, as depicted in Figure 8(c,d) (*p < 0.01*). However, weight loss was not observed in nude mice exposed to CAR-T cells relative to those treated with PBS and untransduced T cells (*p < 0.01*; Figure 8(e)).

**Figure 9.**
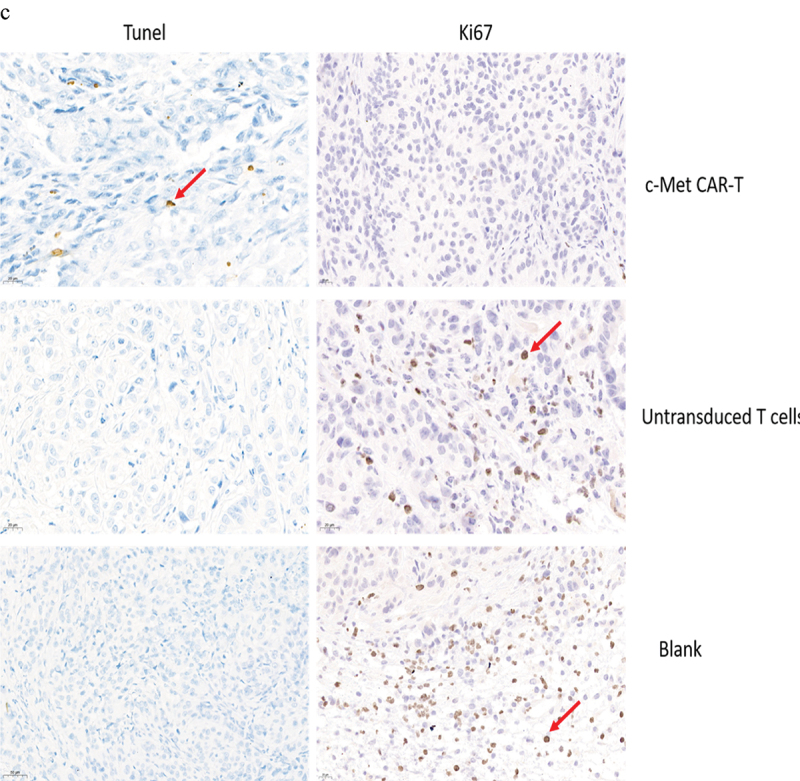
Continued.

The nude mice were euthanized on day 30. The H&E staining of the spleen, liver, lung, and kidney indicated that c-Met CAR-T cells did not cause necrosis in the healthy tissues of the vital organs of the mice (Figure 9(a)). In addition, we performed immunohistochemical (IHC) analysis of the xenograft, and the data indicated that the number of c-Met positive A549 cells in the xenograft of nude mice exposed to c-Met CAR-T cells was considerably decreased compared to that in the blank and T cell groups (Figure 9(b)). The expression of Ki67 in the tumor tissue was identified via IHC analysis. In tumor tissue exposed to c-Met CAR-T cells, a decreased level of Ki67. And A549 cells apoptosis was detected by the Tunel method, result revealed positive staining of A549 cells only after c-Met CAR T treatment , as indicated in Figure 9(c).
**Figure 9.**
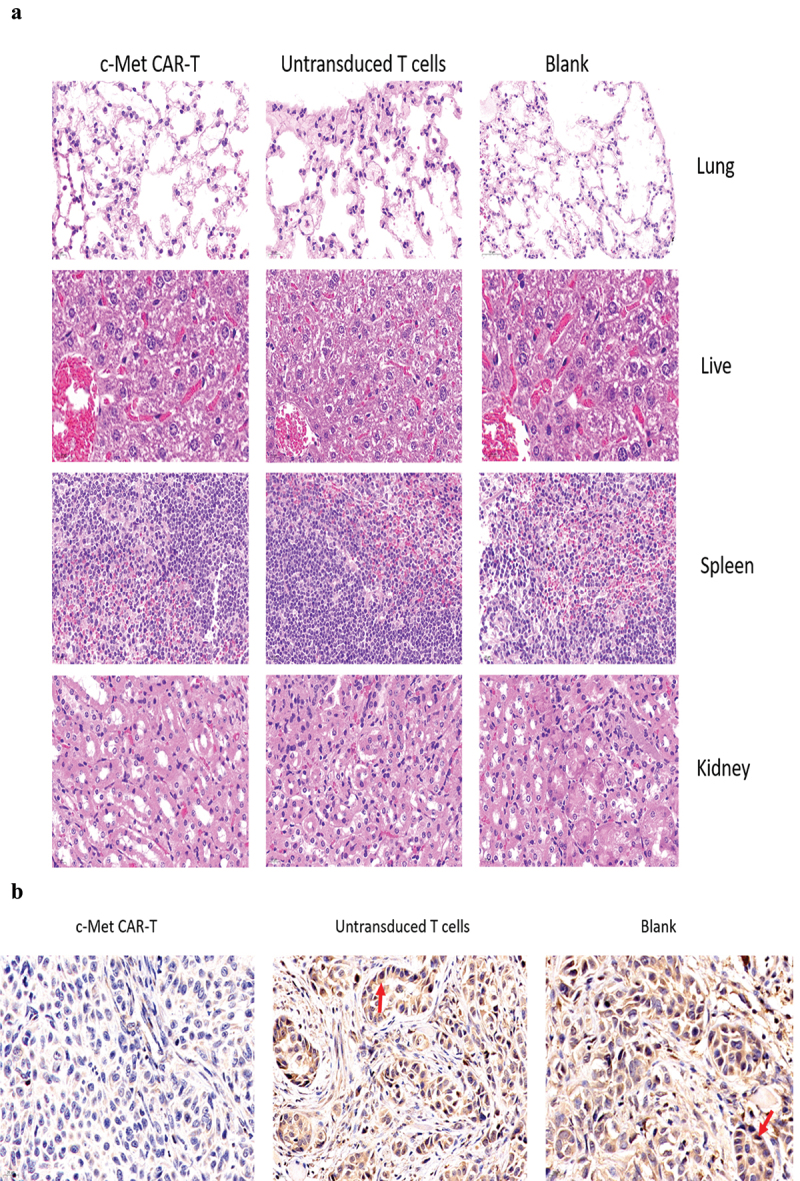
HE and immunohistochemical staining of internal organ and subcutaneous transplanted tumors was performed in 3 groups. Internal organ and Tumor specimens were collected on day 30; (a) HE staining was performed to evaluate the pathological changes of lung, liver, kidney and spleen tissue from the 3 group (×400); (b)c-Met was mainly located in the cytoplasm and on the surface of A549 cells. Strong and diffuse expression of c-Met in Blank group and untransduced T cells group (Cells membrane and cytoplasm stained in yellow brown were the positive pattern, red arrow). c-Met CAR-T group with poor expression of c-Met(× 400); (c) IHC staining of Ki-67 in 3 groups of A549 cell xenograft tumors; The morphological change of apoptosis was observed by TUNEL method(Cells nucleus stained in yellow brown were the positive pattern, red arrow). (×400).

## Discussion

CAR-T cells therapy can target tumors precisely through genetic modification and has achieved promising results in recent years. This strategy is regarded as a breakthrough approach in tumor therapy. CAR-T cells can accurately bind to target cells, activate cytotoxic T lymphocytes through signal transduction, and destroy tumor cells directly or indirectly by releasing perforin and granulase, activating the Fas-FasL pathway, and secreting cytokines [33]. In comparison to conventional T cells, CAR-T cells can use their receptors to specifically attack tumor cells that express specific antigens without the need for MHC. As a result, CAR-T cells are recognized for their ability to kill tumor cells that lack MHC molecules [34]. Additionally, all post-second-generation CAR-T cells express the co-stimulatory signal domains CD28 and/or 4–1BBL, which act as a second signal for T cell activation, allowing for faster and stronger signaling activity, as well as prolonging T cell half-life and retaining the anticancer effect. CAR-T cells have good homing and tissue penetration compared to antibody drugs [35], and CAR-T cells can proliferate, maintain therapeutic doses in vivo, and even generate memory T cells in response to tumor-specific antigens [36].

Direct tumor cell elimination is mediated by CD8^+^ CAR-T cells in particular. Th-cells (CD4^+^ T helper cells) have been identified as an effective and clinically significant T cell subgroup [37]. CD4^+^ CAR-T cells have been shown to exhibit cytotoxic potential equivalent to cytotoxic CD8^+^ CAR-T cells. In this study, we analyzed untransfected T cells, CD19 CAR-T, and c-Met CAR-T subtypes. The underlined cells had identical activation and culture conditions. The ScFv fragment was the only difference between CD19 CAR and c-Met CAR, and indeed caused a difference in the proportion of T cell subtypes after lentivirus infection. However, this judgment is only based on our test conditions. Furthermore, the differentiation of T cell subtypes has not been determined at present. Adoptively transplanted T cell subtypes have been identified as one of the most important success criteria for effective immunotherapy. T cell subtype regulation could be able to boost therapeutic CAR-T efficacy. Based on the current research, retronectin-based T cell activation can increase the amount of cytotoxic CD8^+^ T cells and possibly shift the CD4:CD8 ratio toward 1:1 [38]. And cytokines such as IL-2, IL-7, and IL-15 may affect the proportion of CD4^+^ CAR-T and CD8^+^ CAR-T [39]. The obtained data revealed that different cultural settings have various impacts on effector functions; however, it’s currently unclear which will be most advantageous in in vivo experiments. To achieve the best possible combination of CAR-T expansion and c-Met selective effector functions, these growth parameters will be utilized to generate c-Met CAR-T cells for our next studies.

In this study, CD19 CAR-T and uninfected T cells uninfected with lentivirus were used as effector cells in the control group to confirm the destroying efficiency of c-Met CAR-T cells on c-Met positive cells (target cells). When the effect-target ratio was higher than 5:1, the destroying efficiency of c-Met CAR against A549 cells was significantly elevated as compared with that of CD19 CAR-T and uninfected T cells. But there is no such effect on Non-target cells killing. When the effect-target ratio was 5:1, the content of cytokines in the supernatant of c-Met CAR-T cells was seeded with c-Met positive target cells was significantly increased. In addition, a non-target group was set to confirm that the destroying efficiency of c-Met CAR-T cells against target cells was consistent with the positive expression of c-Met. The results showed that the cytotoxic effect of c-Met CAR-T cells to A2780 cells without c-Met expression was much lower than that of A549 cells. Furthermore, no differences were observed among the c-Met CAR-T cells CD19 CAR-T cells and T cells, indicating that c-Met CAR-T cells can accurately recognize and attack target cells as well as c-Met CAR-T cells. In the nude mice xenotransplantation model, c-Met CAR-T cells effectively inhibit xenograft tumor growth, with no obvious negative impact during treatment and no effect on nude mice.

Taken together, the obtained data revealed that c-Met CAR-T cells may serve as a highly effective treatment for NSCLC and have a potential application in the treatment of NSCLC. However, the exact functional mechanism of c-Met CAR-T cell immunotherapy remains to be evaluated in in vivo experiments. Moreover, the limitations of the tumor microenvironment, such as immunosuppression, homing, and maintenance difficulties, need to be addressed in subsequent studies.

## Conclusion

In the current study, a second-generation c-Met CAR molecule was designed. c-Met CAR-T cells with stable expression of CAR sequences were successfully constructed. In vitro experiments have shown that c-Met CAR-T cells can target c-Met positive NSCLC cells, while enhancing proliferation and releasing high levels of cytokines, with the specific killing activity of tumor cells. c-Met CAR-T cells can considerably attenuate the growth of subcutaneous grafted tumors of luciferase engineered A549 cells, however, off-target toxicity was not observed in healthy organs.

## Supplementary Material

Supplemental Material

## Data Availability

Please contact the correspondence author for the data request.

## Animal feeding and environmental conditions

All the experimental mice were raised in the SPF level of Animal Experiment Center of Bengbu Medical College . They were alternately illuminated for 12h per day, with temperature of 24± 2°C, humidity of 40%~70%, and free feed and water. After the treatment, all mice were anesthetized with 4% chloral hydrate by intraperitoneal injection.

At the end of the experiment, the mice were killed by cervical dislocation
