# Supplementary figures and images for: c-Met specific CAR-T cells as a targeted therapy for non-small cell lung cancer cell A549
Source: Bioengineered. 2022 Apr 4;13(4):9232–48. doi: 10.1080/21655979.2022.2058149 (PMC9161852; doi:10.1080/21655979.2022.2058149)

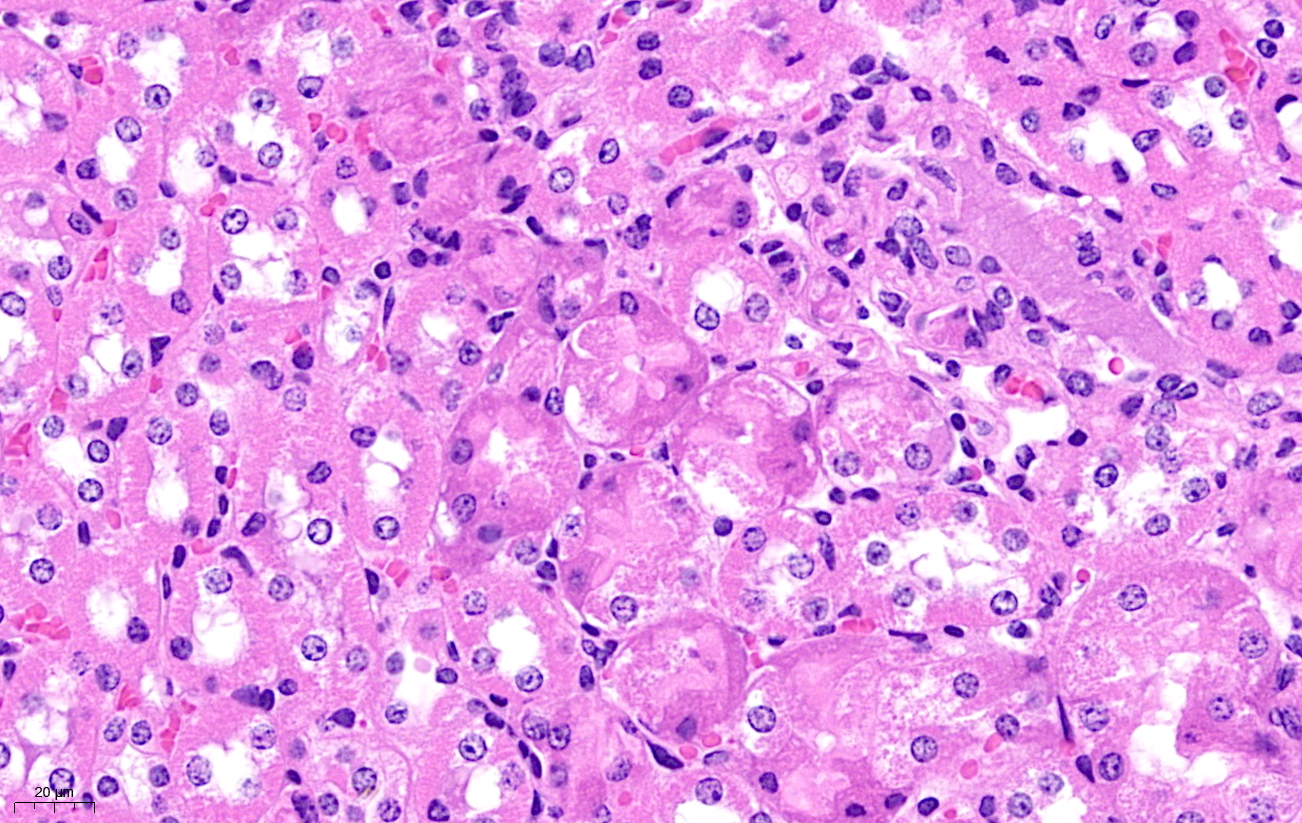

Supplement: Supplemental Material [file KBIE_A_2058149_SM0390.zip › supplementary/Blank group kidned HE staining.jpg]

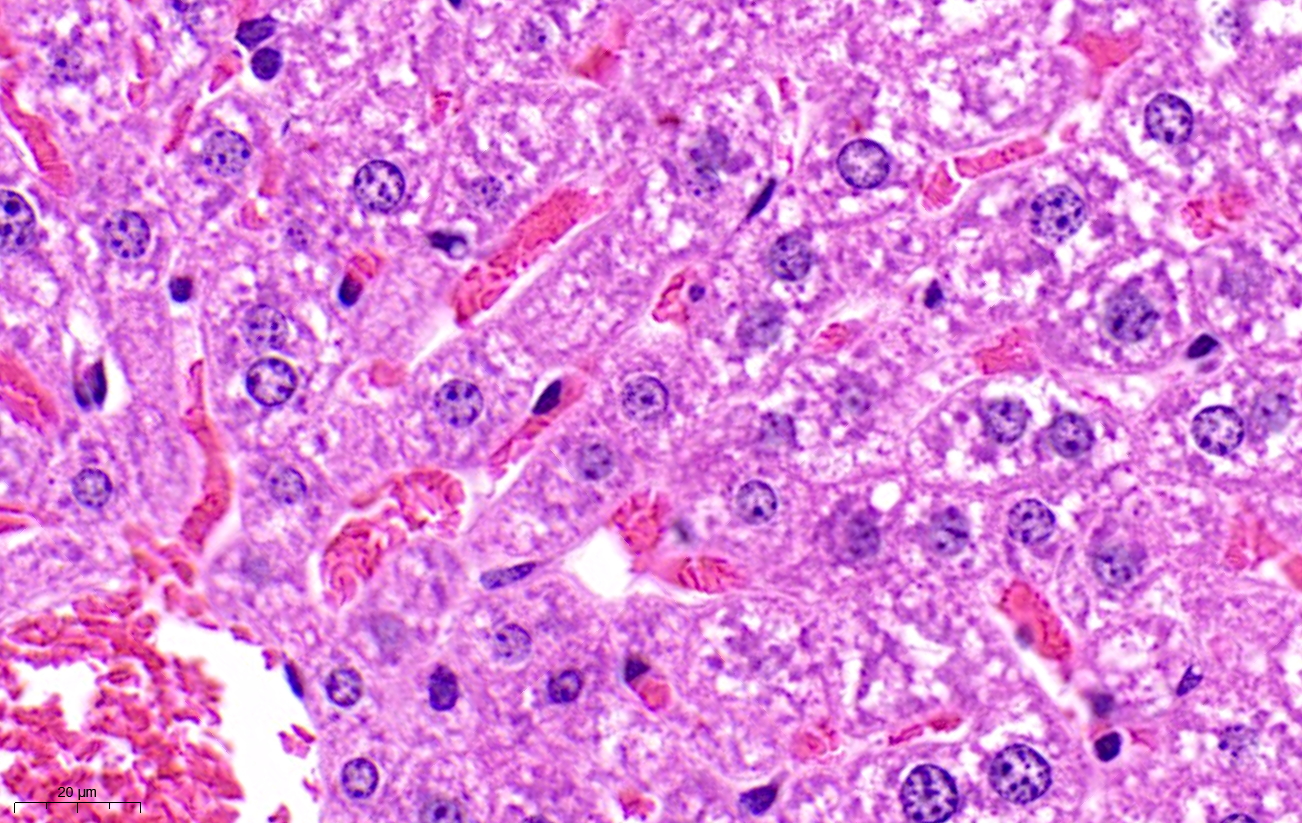

Supplement: Supplemental Material [file KBIE_A_2058149_SM0390.zip › supplementary/Blank group live HE staining.jpg]

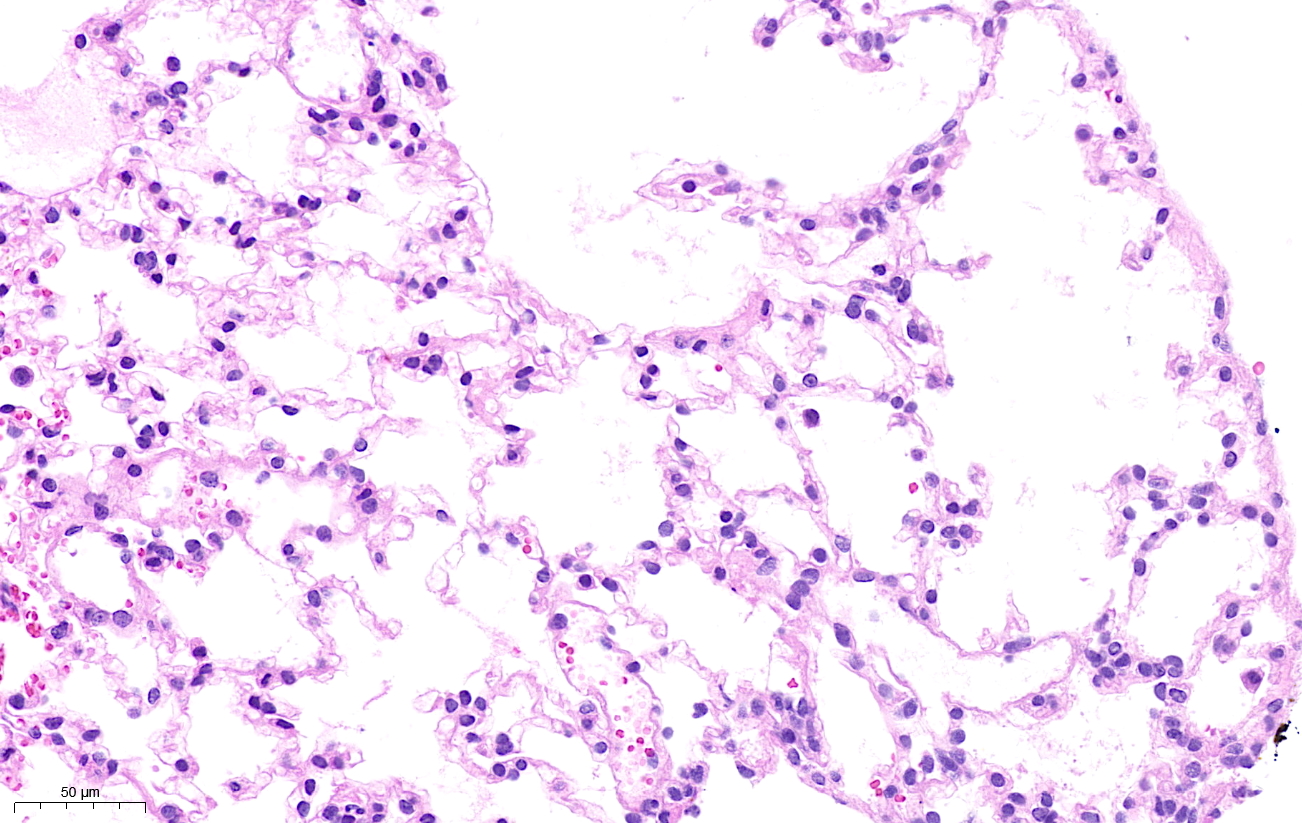

Supplement: Supplemental Material [file KBIE_A_2058149_SM0390.zip › supplementary/Blank group lung HE staining.jpg]

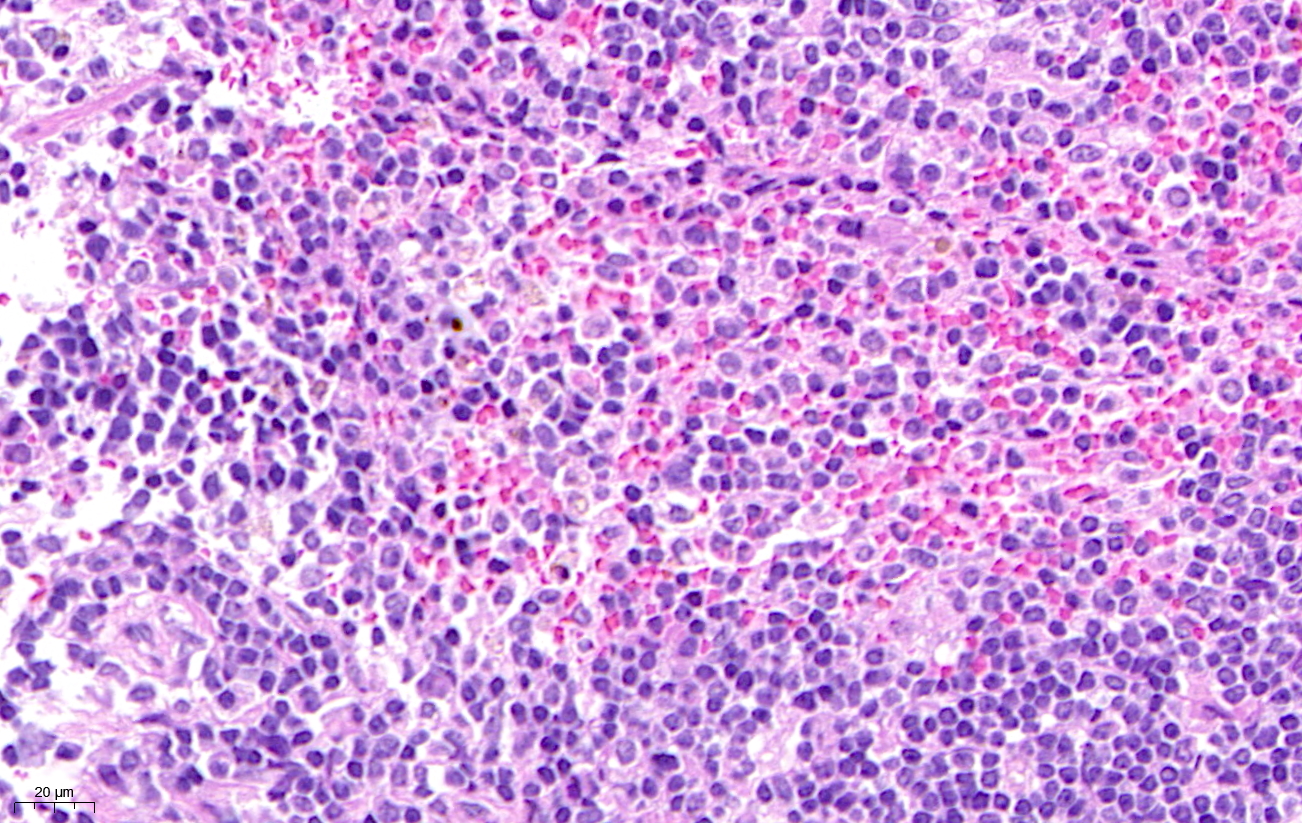

Supplement: Supplemental Material [file KBIE_A_2058149_SM0390.zip › supplementary/Blank group spleen HE staining.jpg]

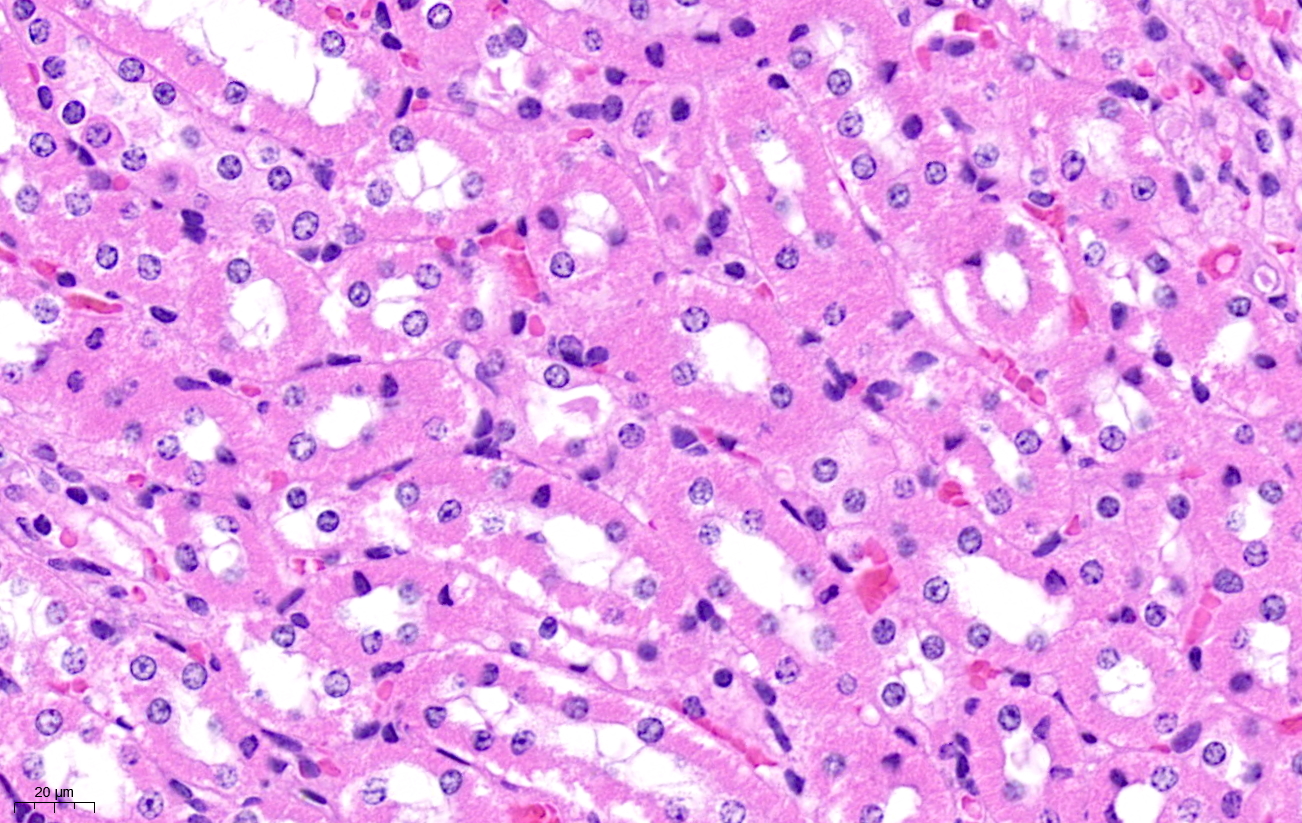

Supplement: Supplemental Material [file KBIE_A_2058149_SM0390.zip › supplementary/c Met CAR T group kidneyHE staining .jpg]

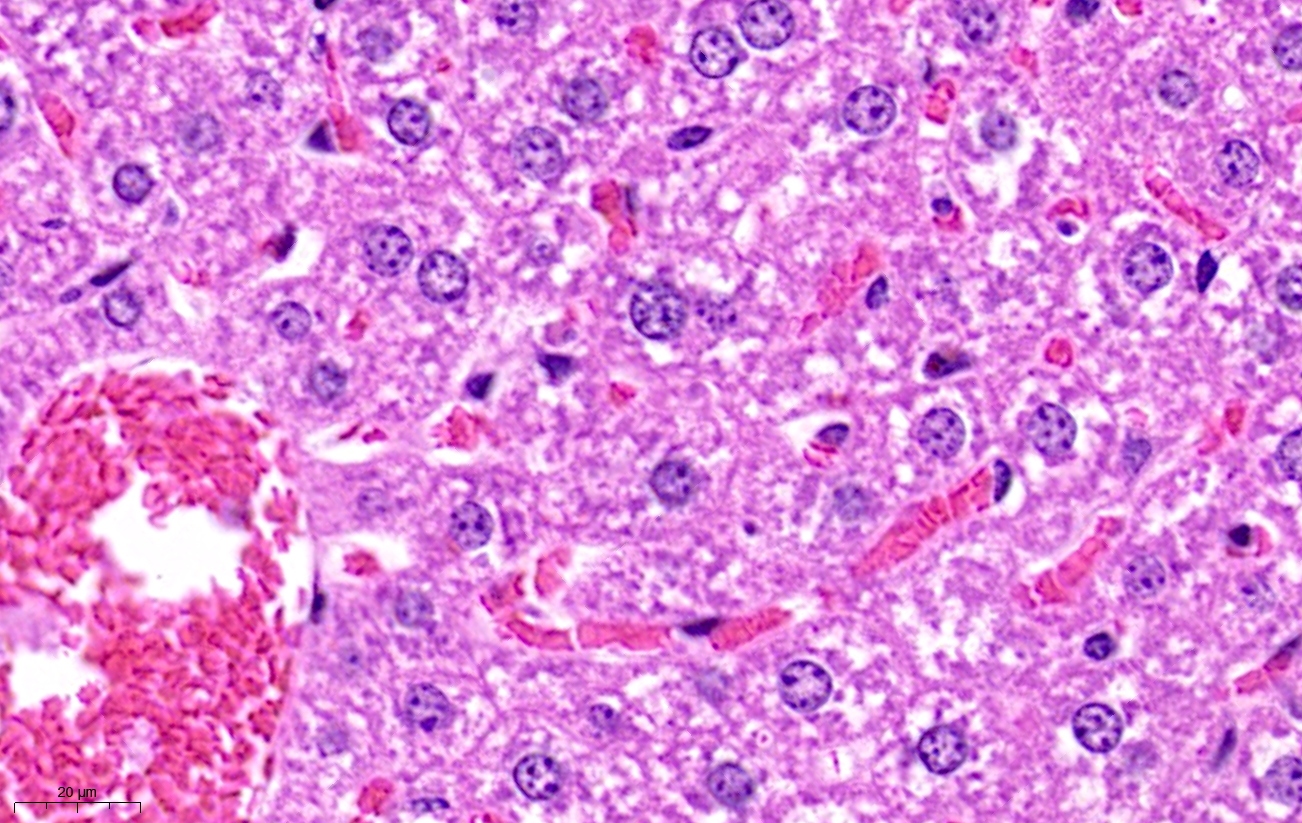

Supplement: Supplemental Material [file KBIE_A_2058149_SM0390.zip › supplementary/c Met CAR T group live HE staining .jpg]

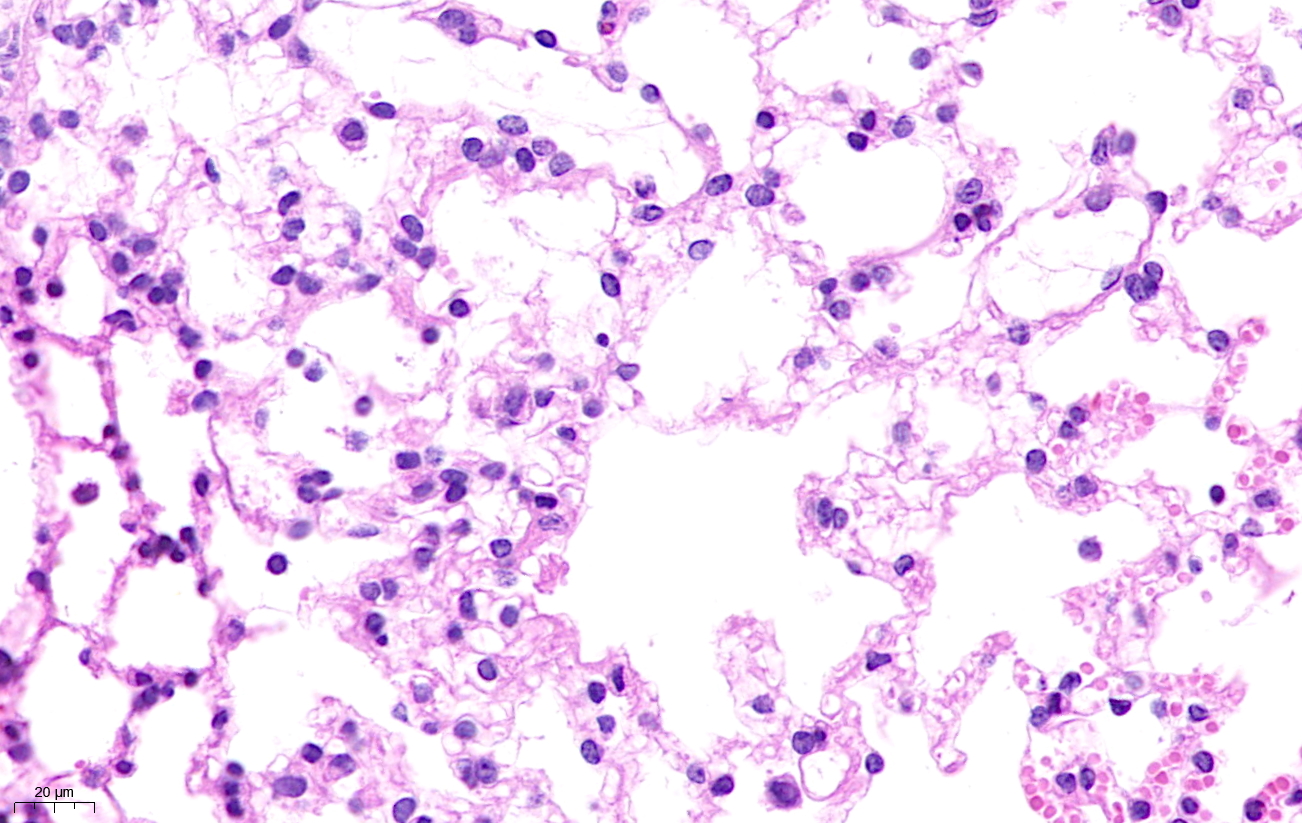

Supplement: Supplemental Material [file KBIE_A_2058149_SM0390.zip › supplementary/c Met CAR T group lung HE staining .jpg]

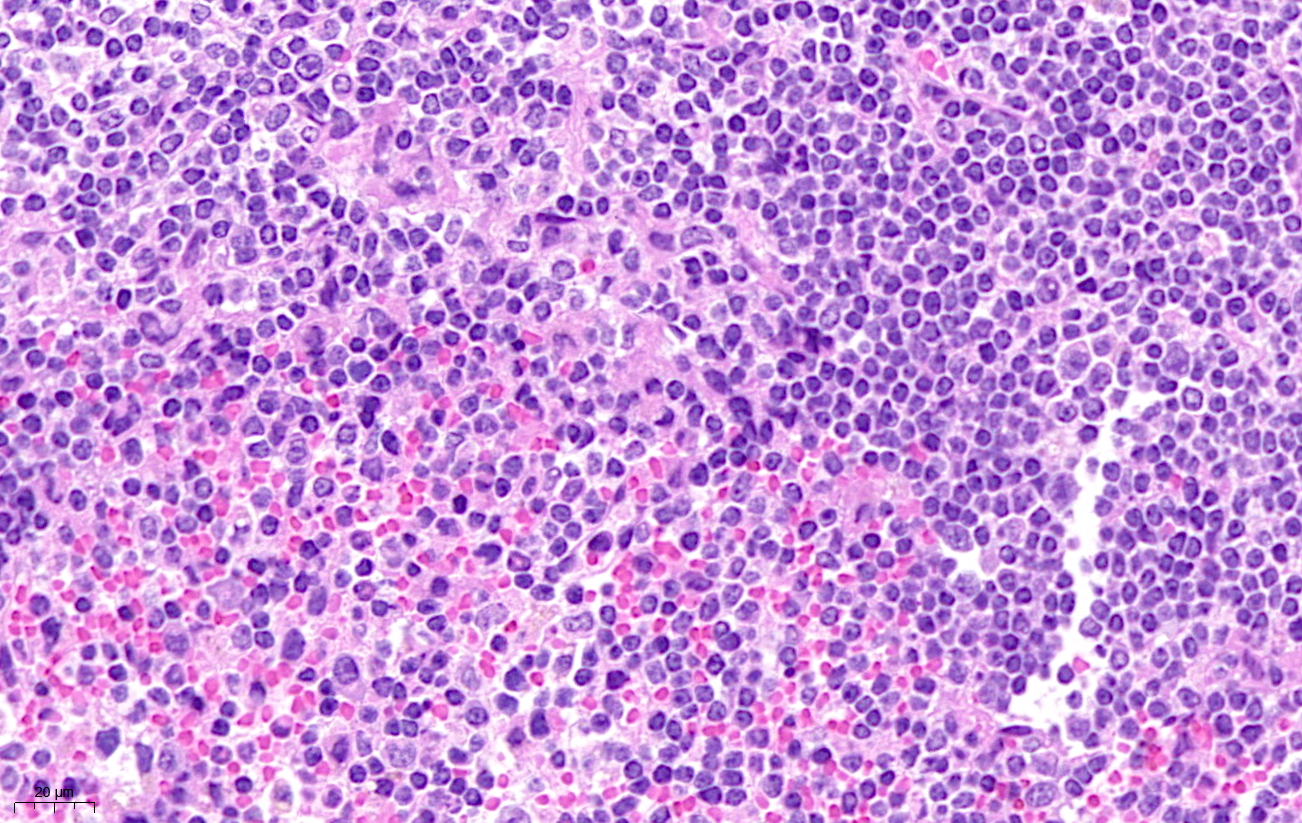

Supplement: Supplemental Material [file KBIE_A_2058149_SM0390.zip › supplementary/c Met CAR T group spleen HE staining.jpg]

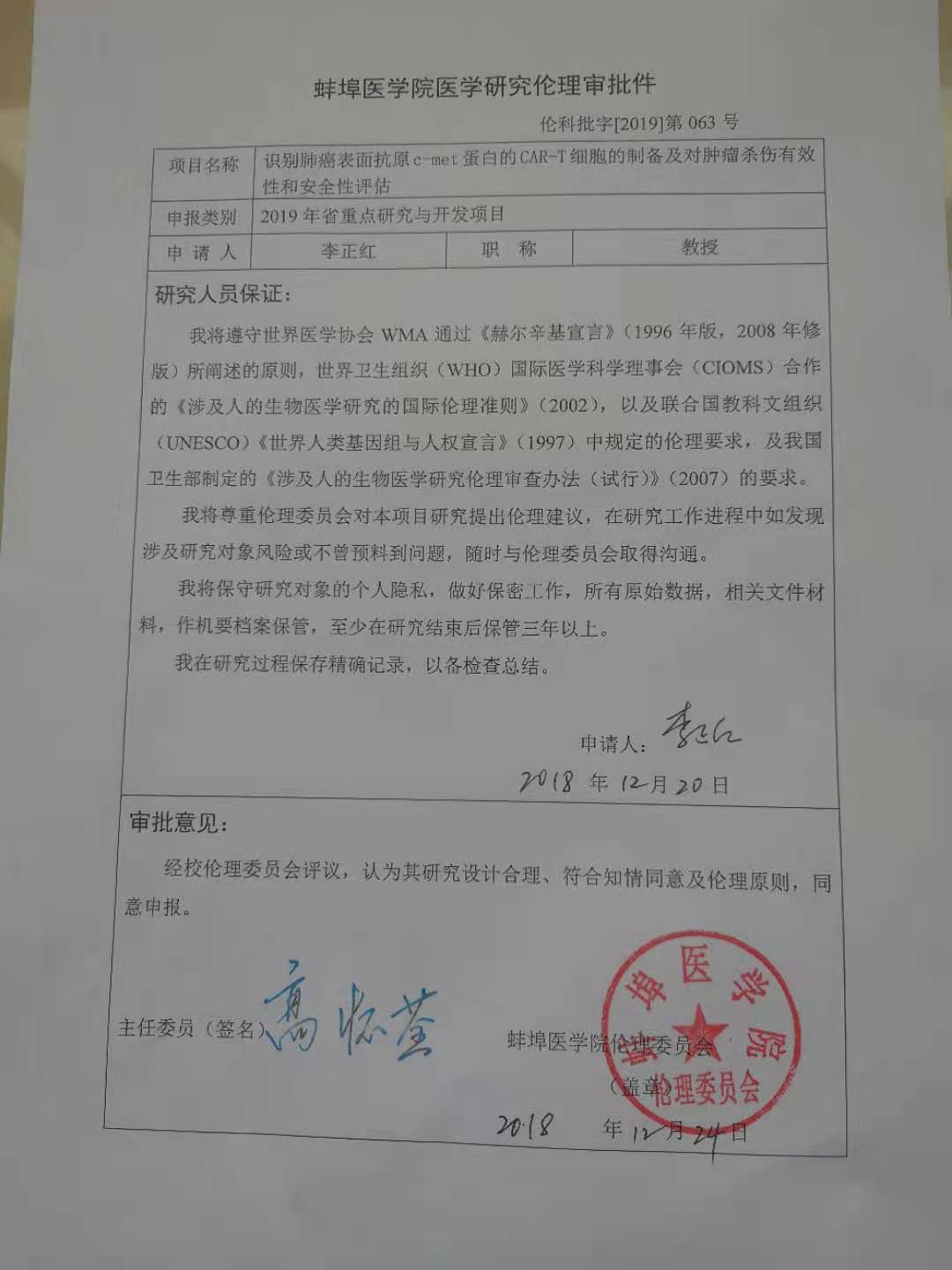

Supplement: Supplemental Material [file KBIE_A_2058149_SM0390.zip › supplementary/ethical approvement 1.jpg]

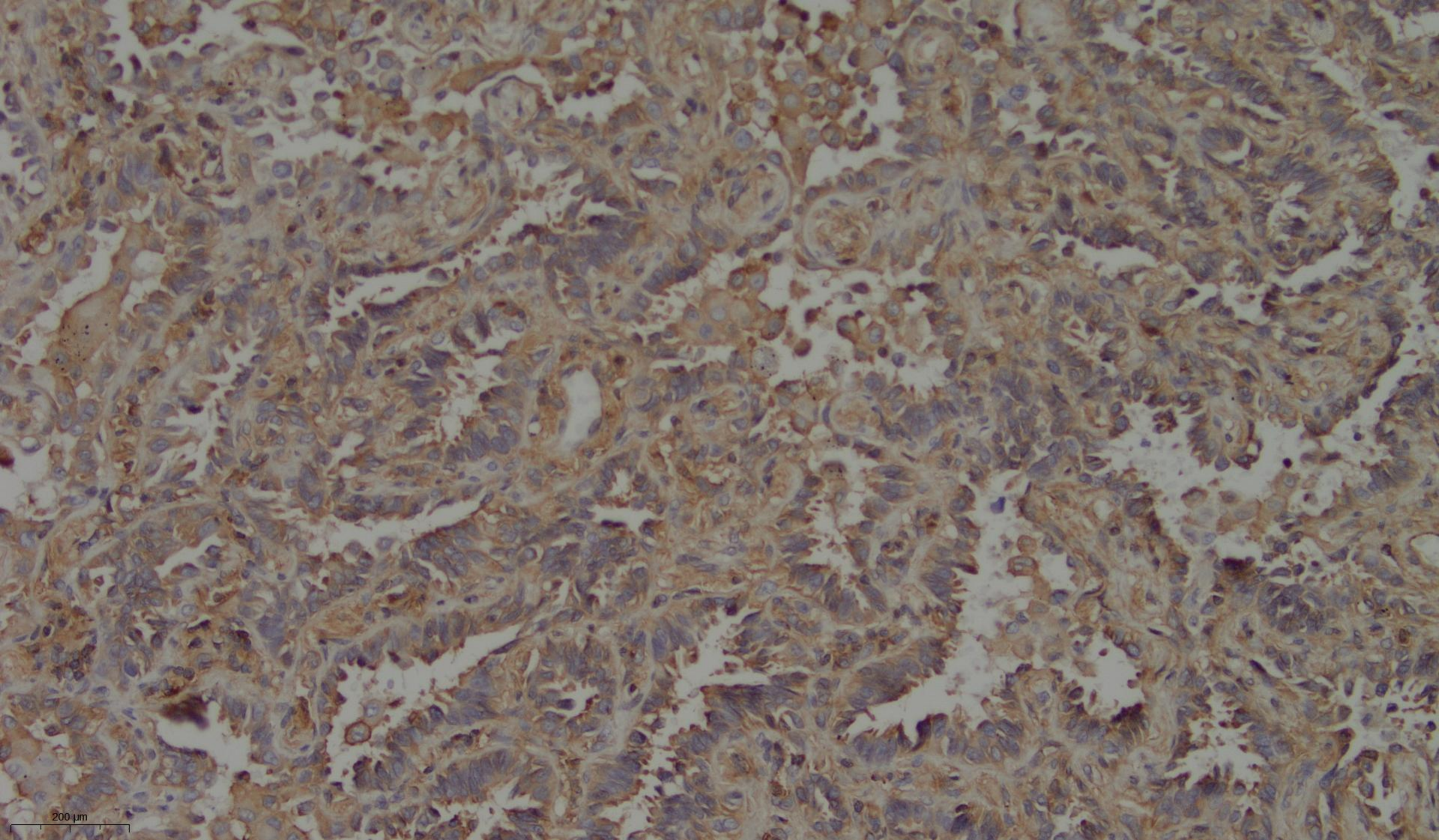

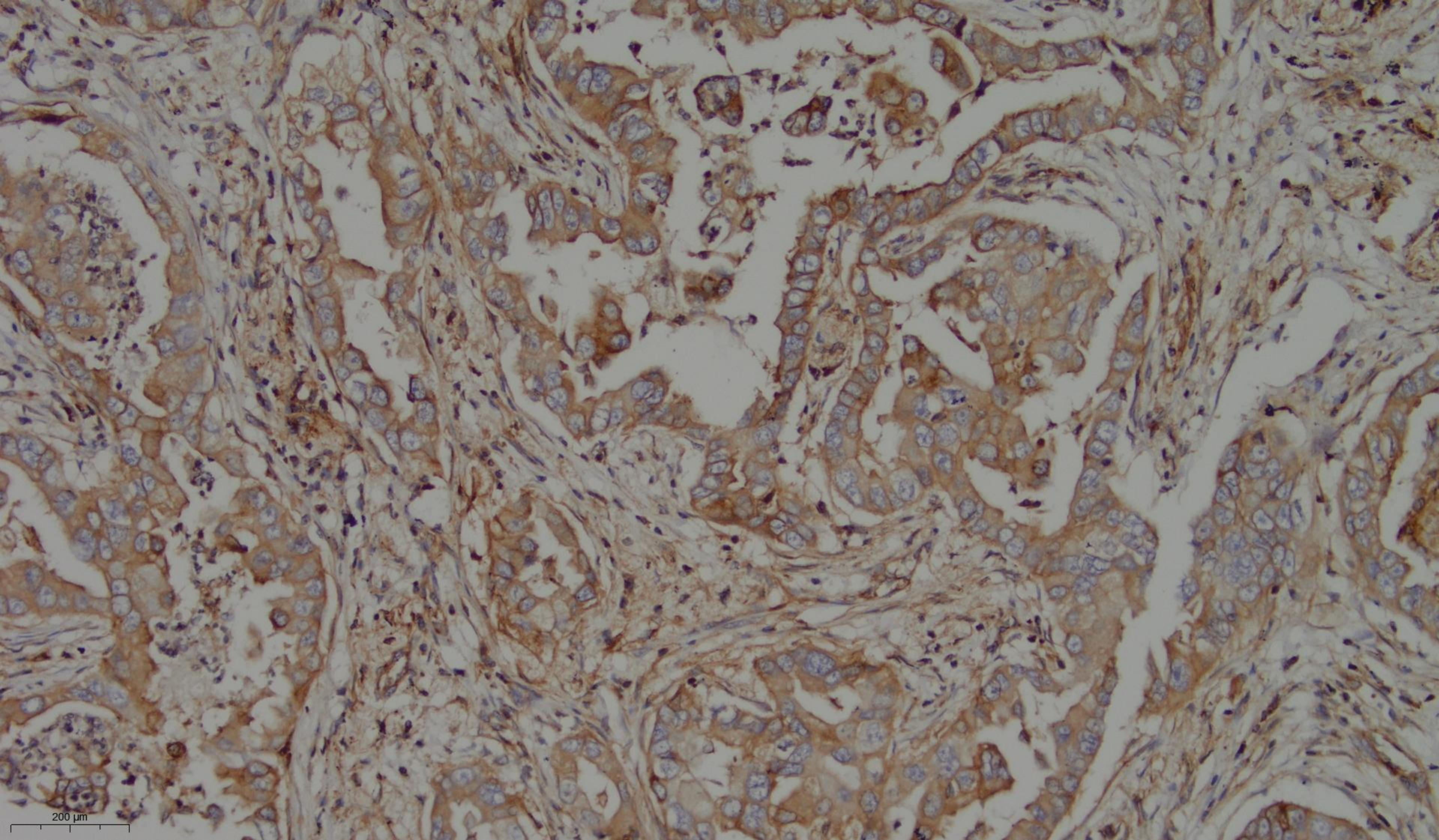

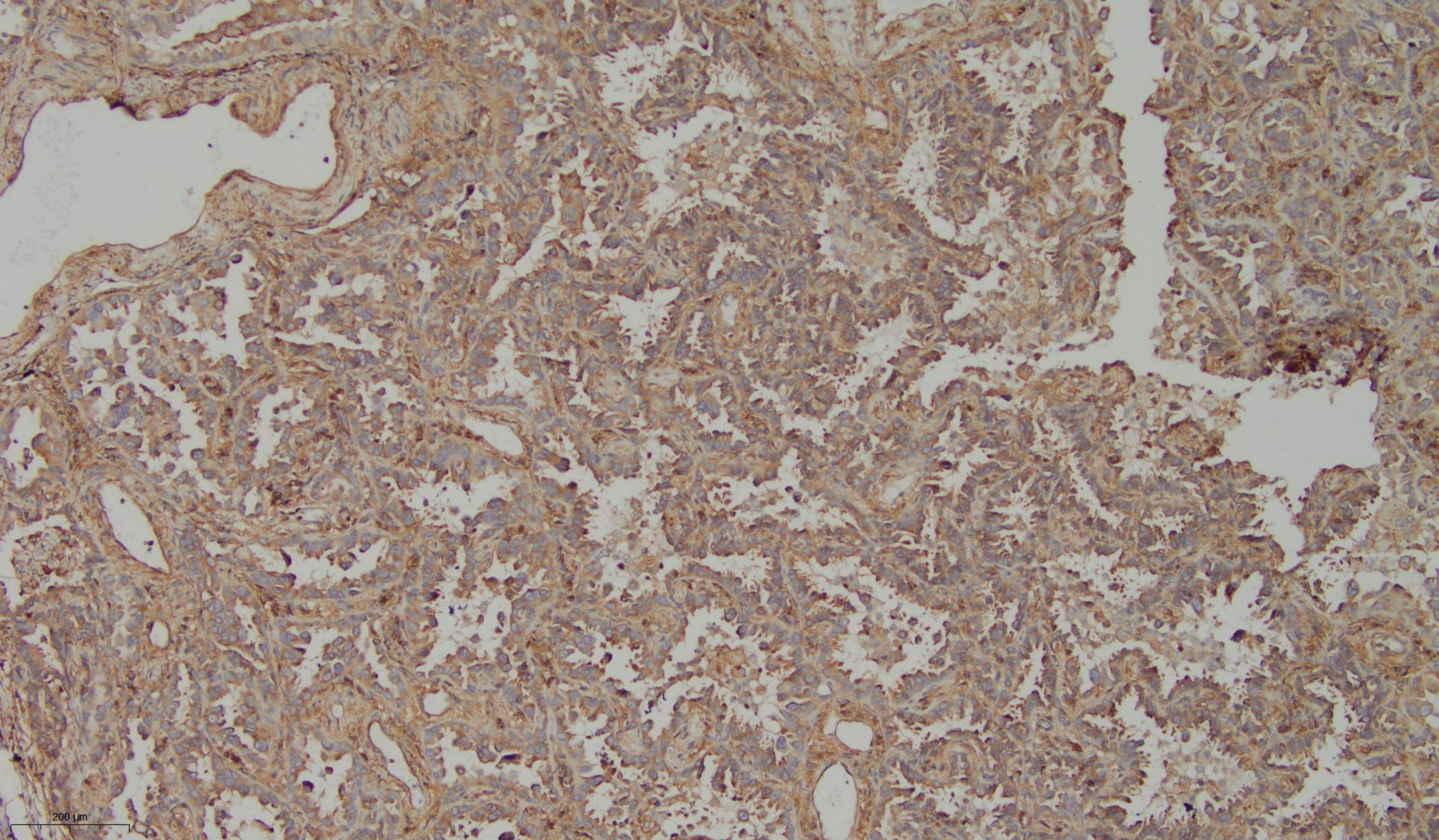

200 μm

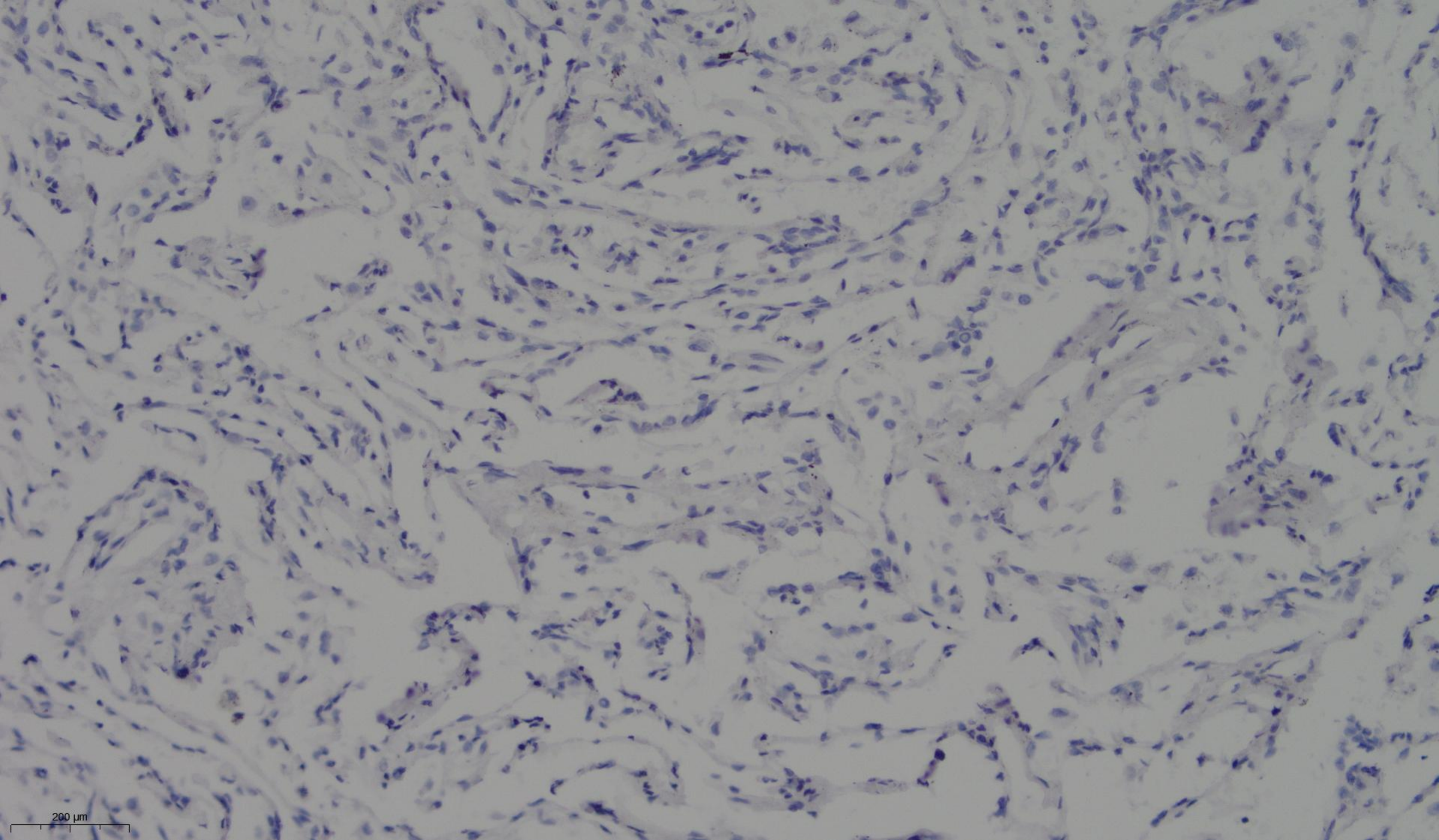

200  $\mu$ m

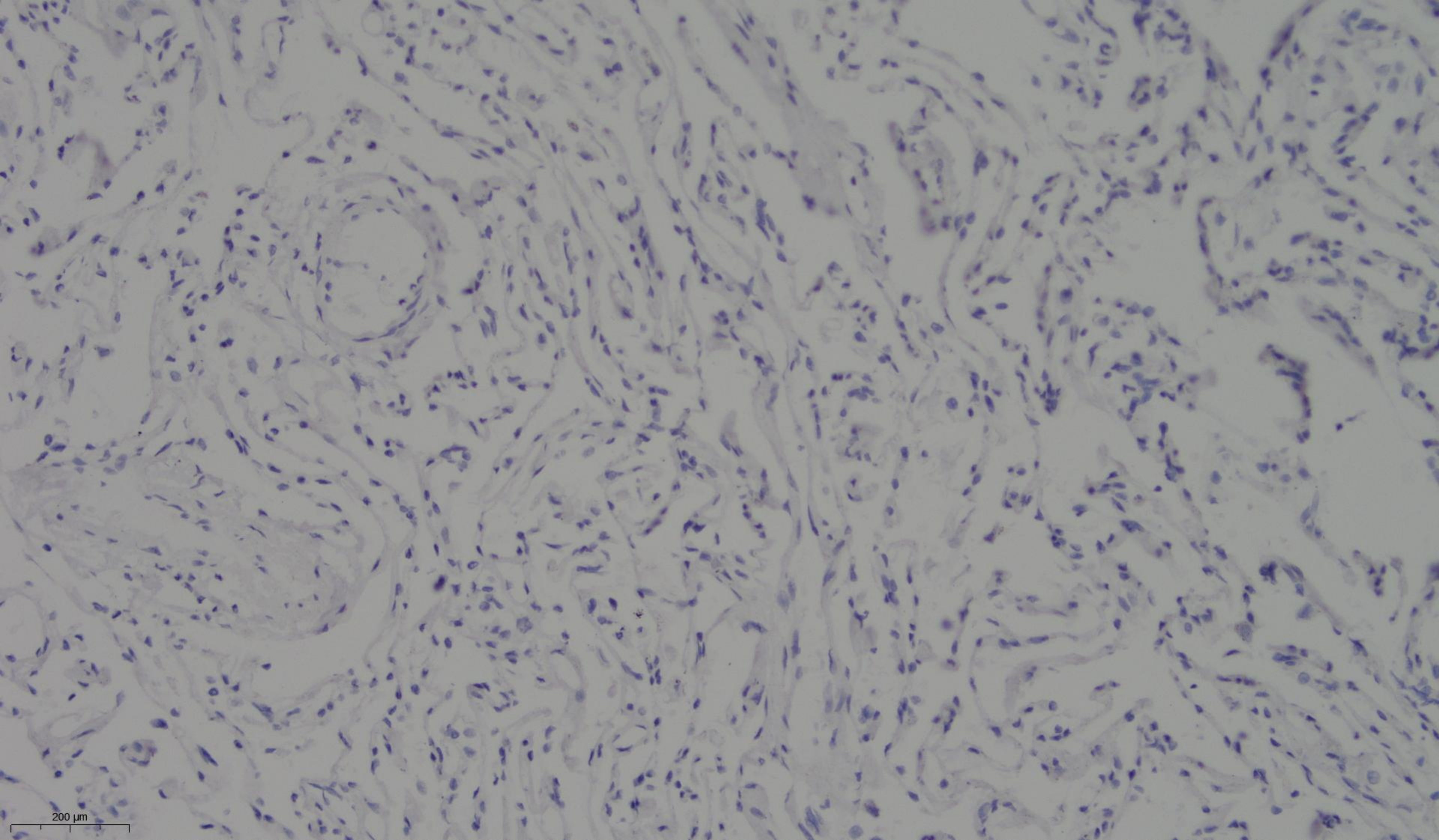

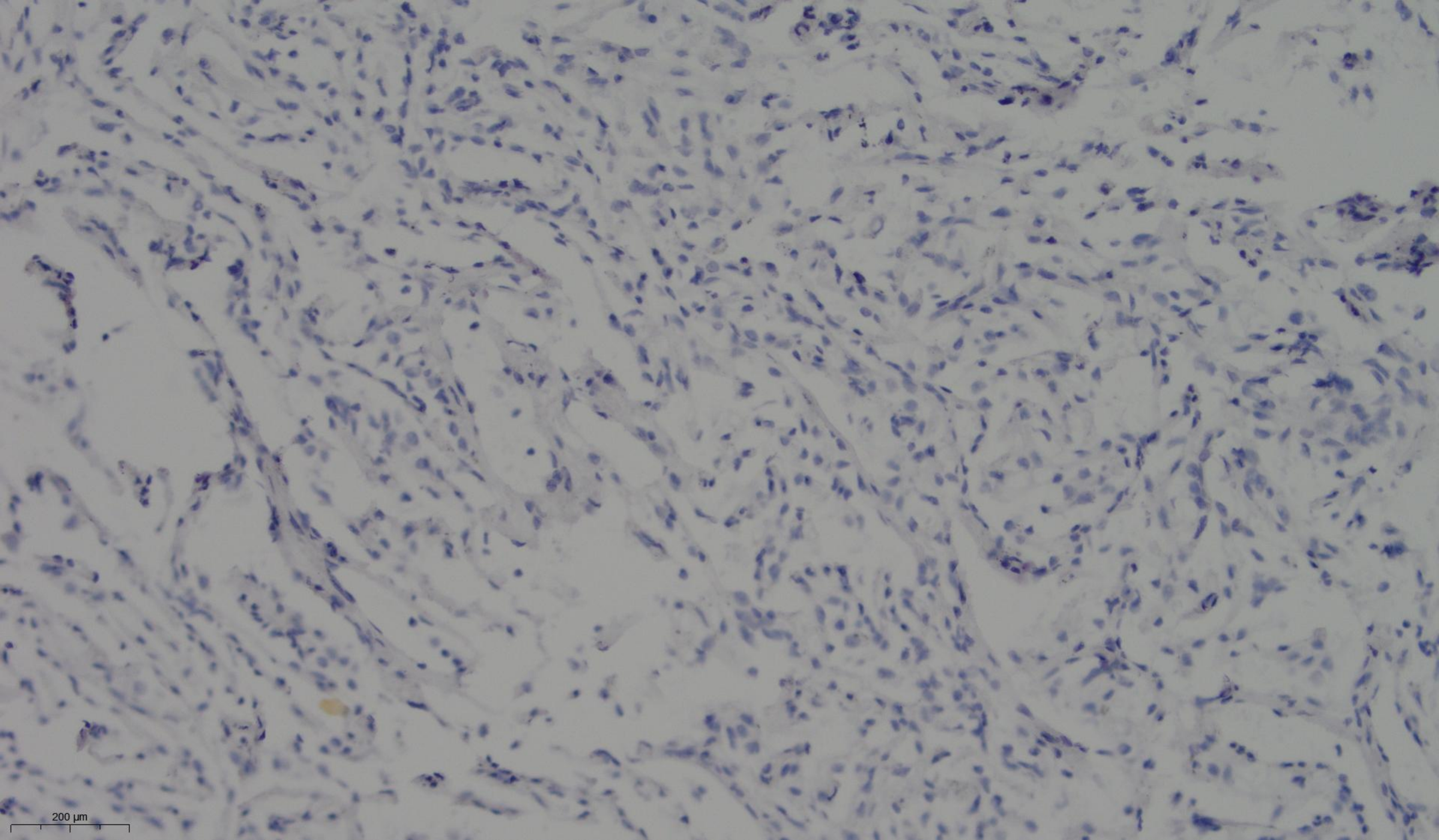

200  $\mu$ m

Supplement: Supplemental Material [file KBIE_A_2058149_SM0390.zip › supplementary/Fig 5 Immunohistochemistry of clinical specimens of NSCLC and adjacent tissues.pdf]

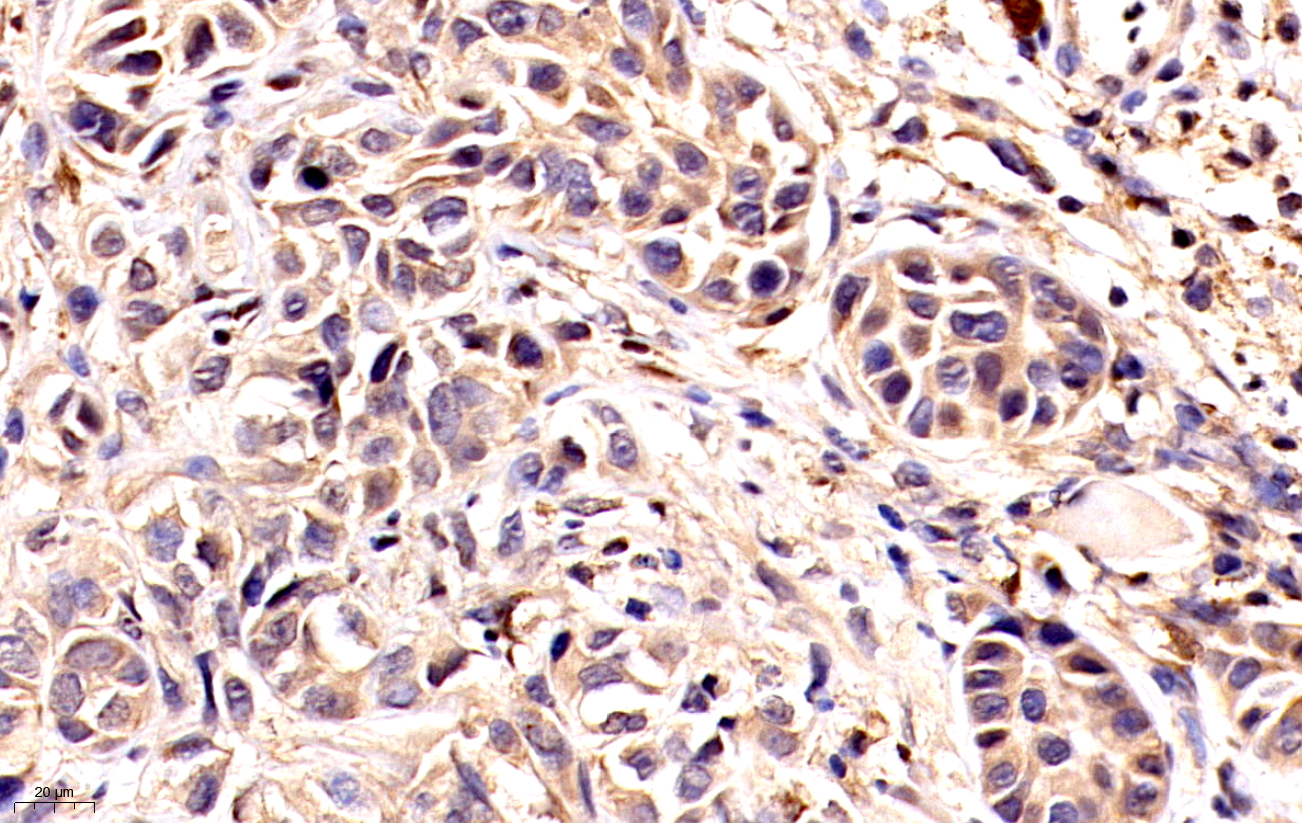

Supplement: Supplemental Material [file KBIE_A_2058149_SM0390.zip › supplementary/Fig9 Blank group c met Immunohistochemistry .jpg]

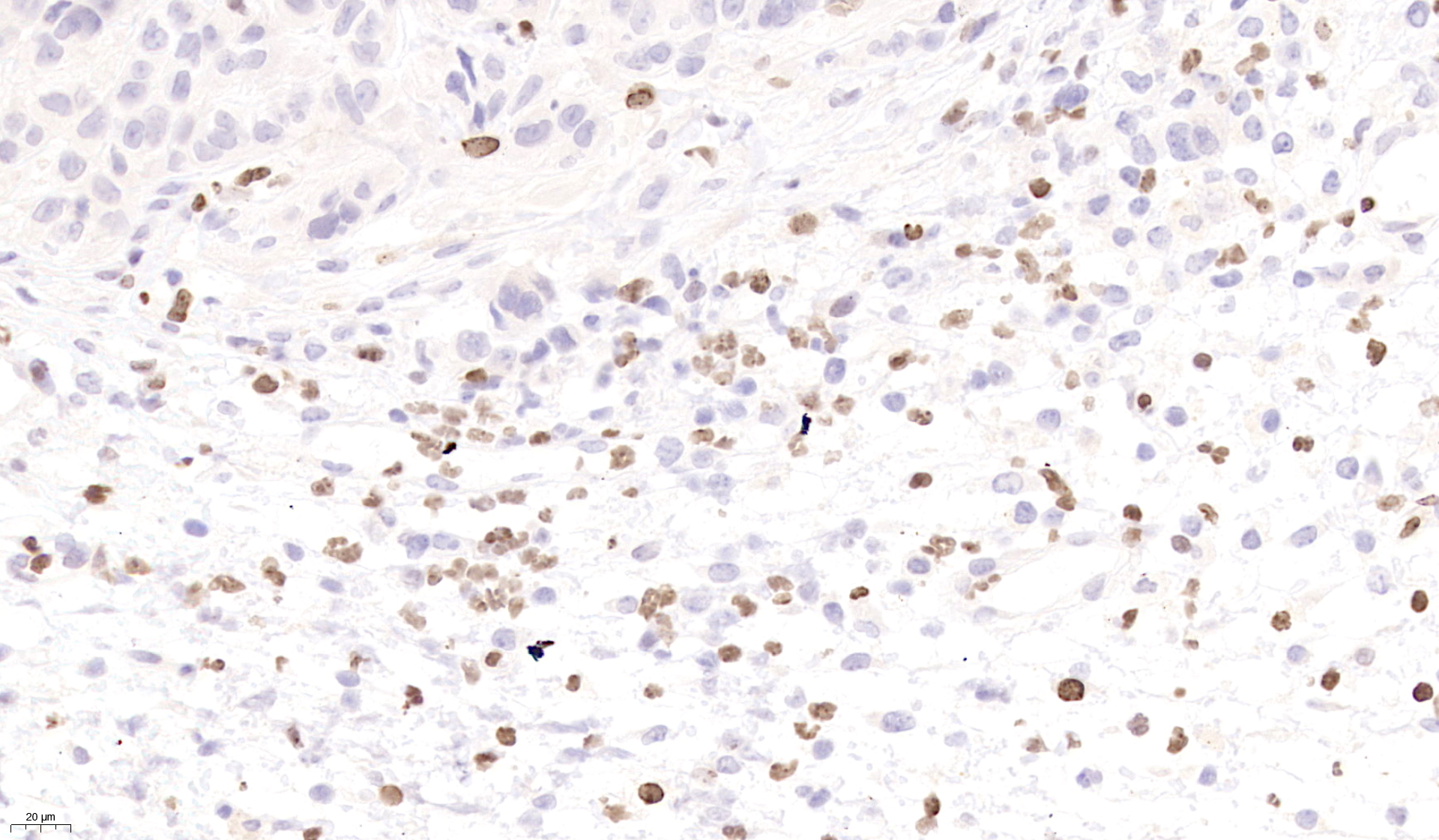

Supplement: Supplemental Material [file KBIE_A_2058149_SM0390.zip › supplementary/Fig9 Blank group KI67 Immunohistochemistry .jpg]

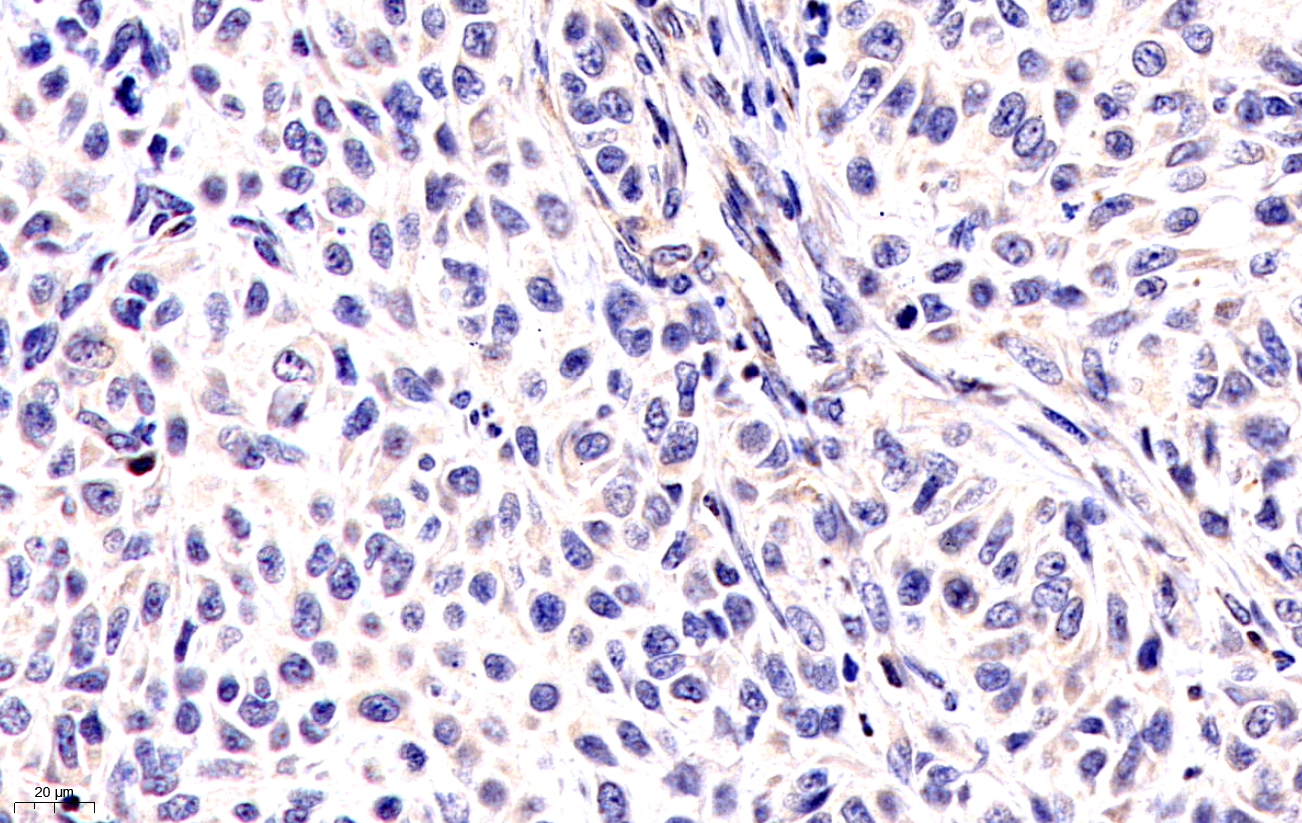

Supplement: Supplemental Material [file KBIE_A_2058149_SM0390.zip › supplementary/Fig9 c Met CAR T group c met Immunohistochemistry .jpg]

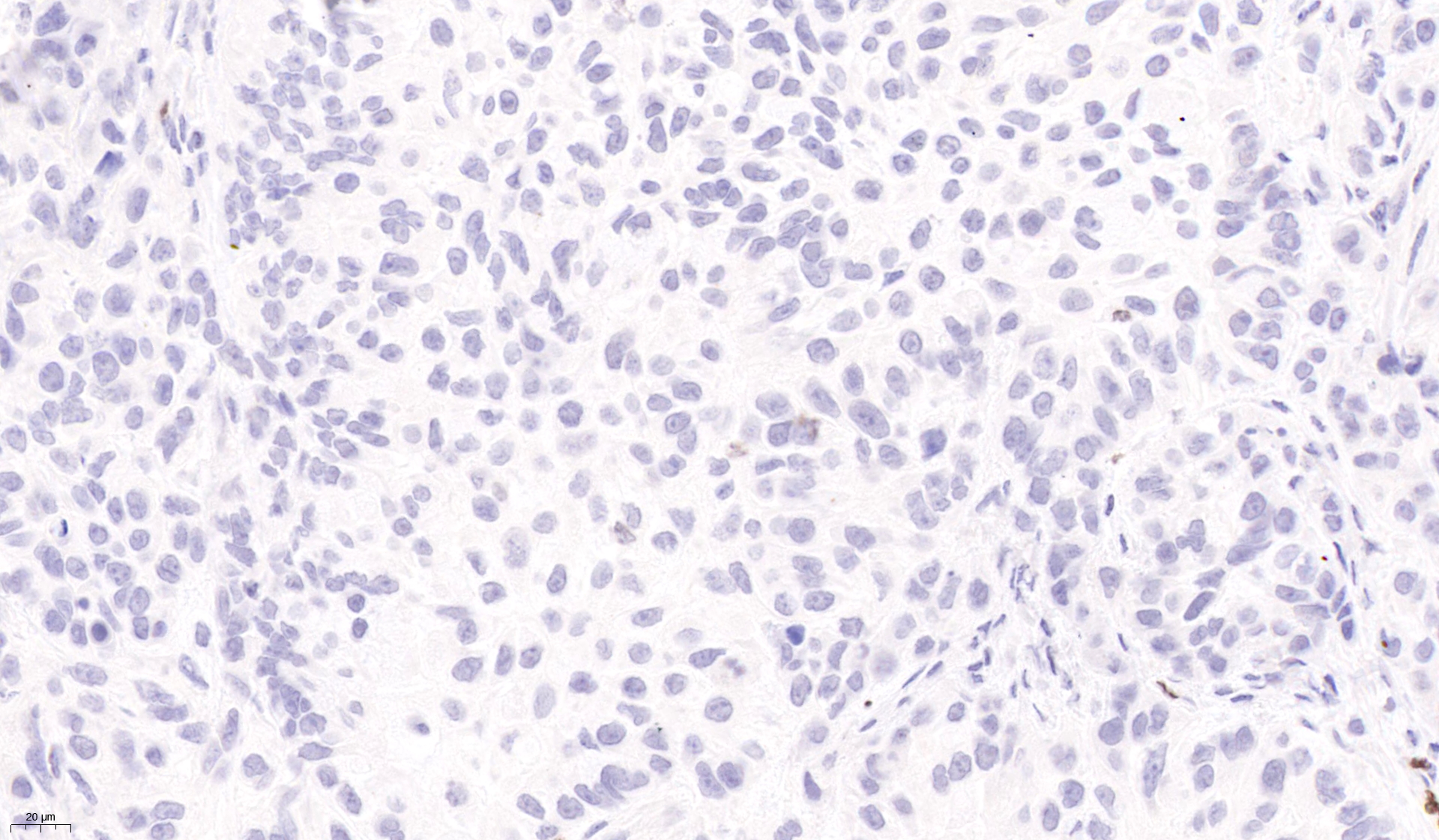

Supplement: Supplemental Material [file KBIE_A_2058149_SM0390.zip › supplementary/Fig9 c Met CAR T group KI67 Immunohistochemistry .jpg]

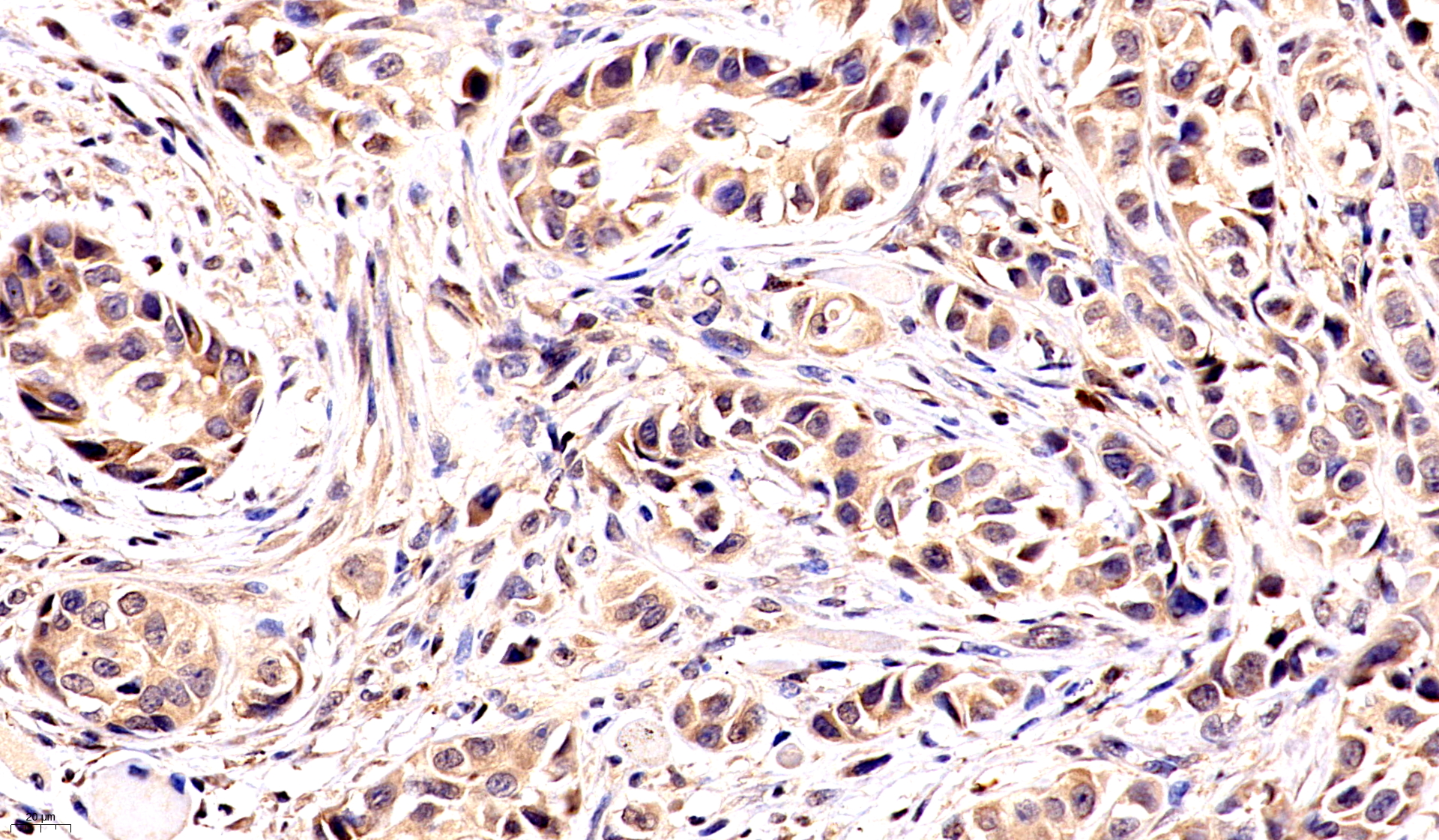

Supplement: Supplemental Material [file KBIE_A_2058149_SM0390.zip › supplementary/Fig9 Untransduced T cells group c met Immunohistochemistry .jpg]

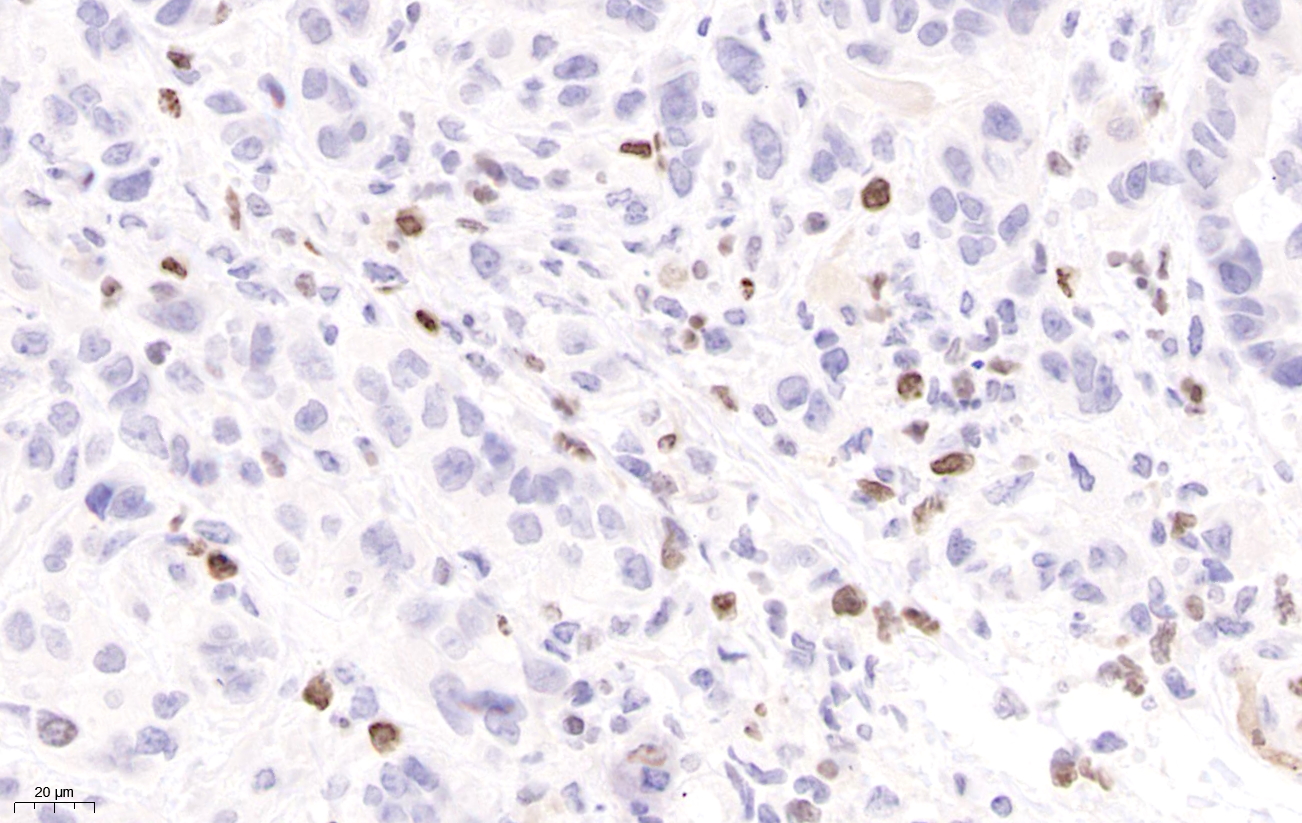

Supplement: Supplemental Material [file KBIE_A_2058149_SM0390.zip › supplementary/Fig9 Untransduced T cells group KI67 Immunohistochemistry .jpg]

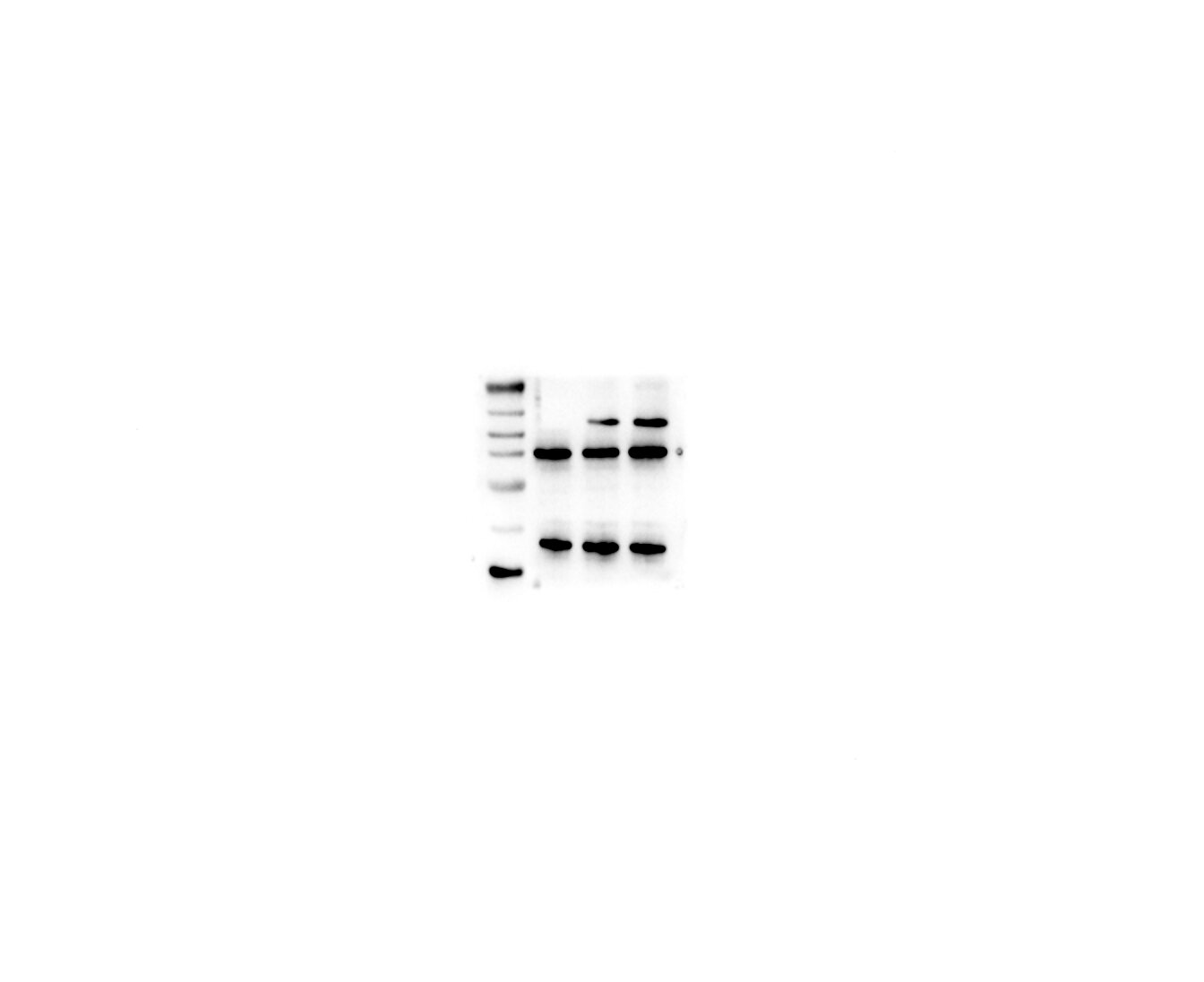

Supplement: Supplemental Material [file KBIE_A_2058149_SM0390.zip › supplementary/original images of western blot.jpg]

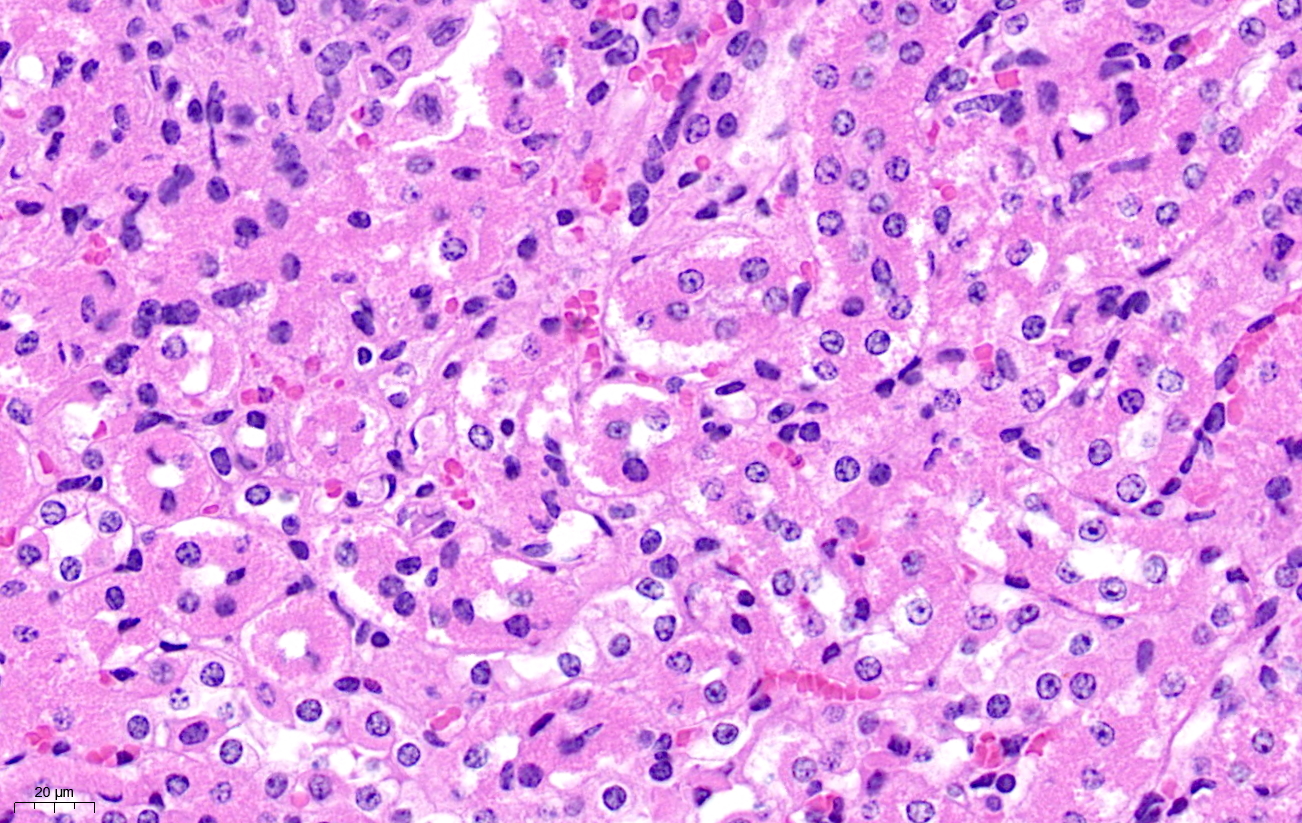

Supplement: Supplemental Material [file KBIE_A_2058149_SM0390.zip › supplementary/Untranstduced T cells group kidney HE staining.jpg]

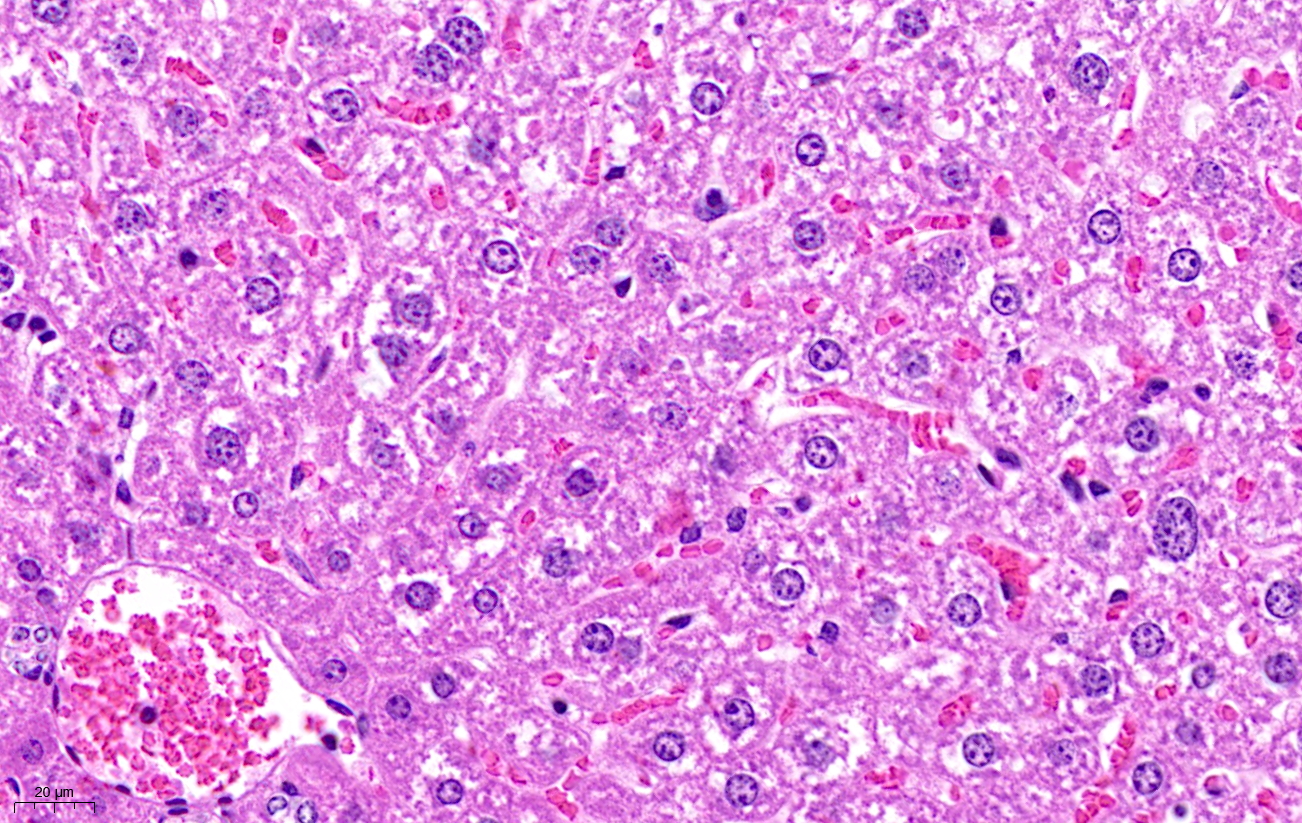

Supplement: Supplemental Material [file KBIE_A_2058149_SM0390.zip › supplementary/Untranstduced T cells group live HE staining.jpg]

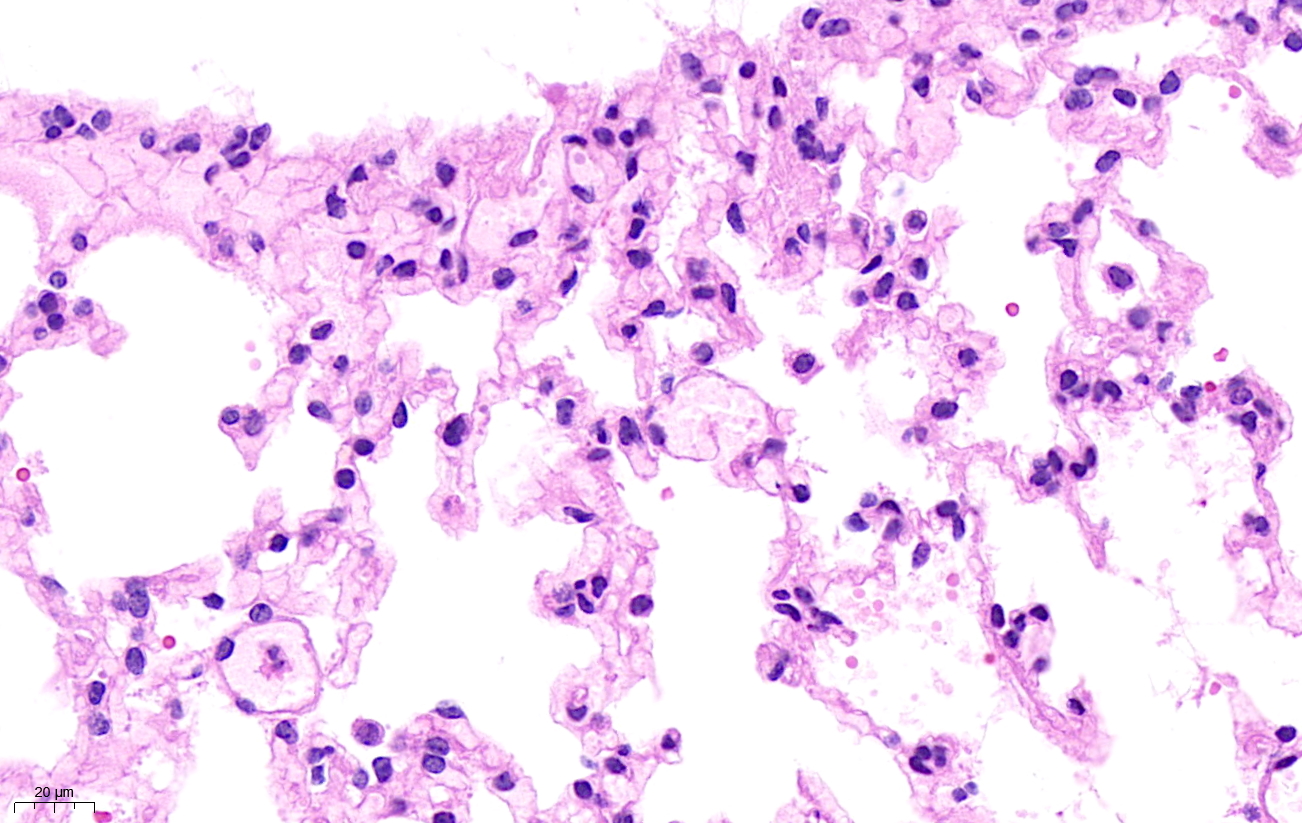

Supplement: Supplemental Material [file KBIE_A_2058149_SM0390.zip › supplementary/Untranstduced T cells group lung HE staining.jpg]

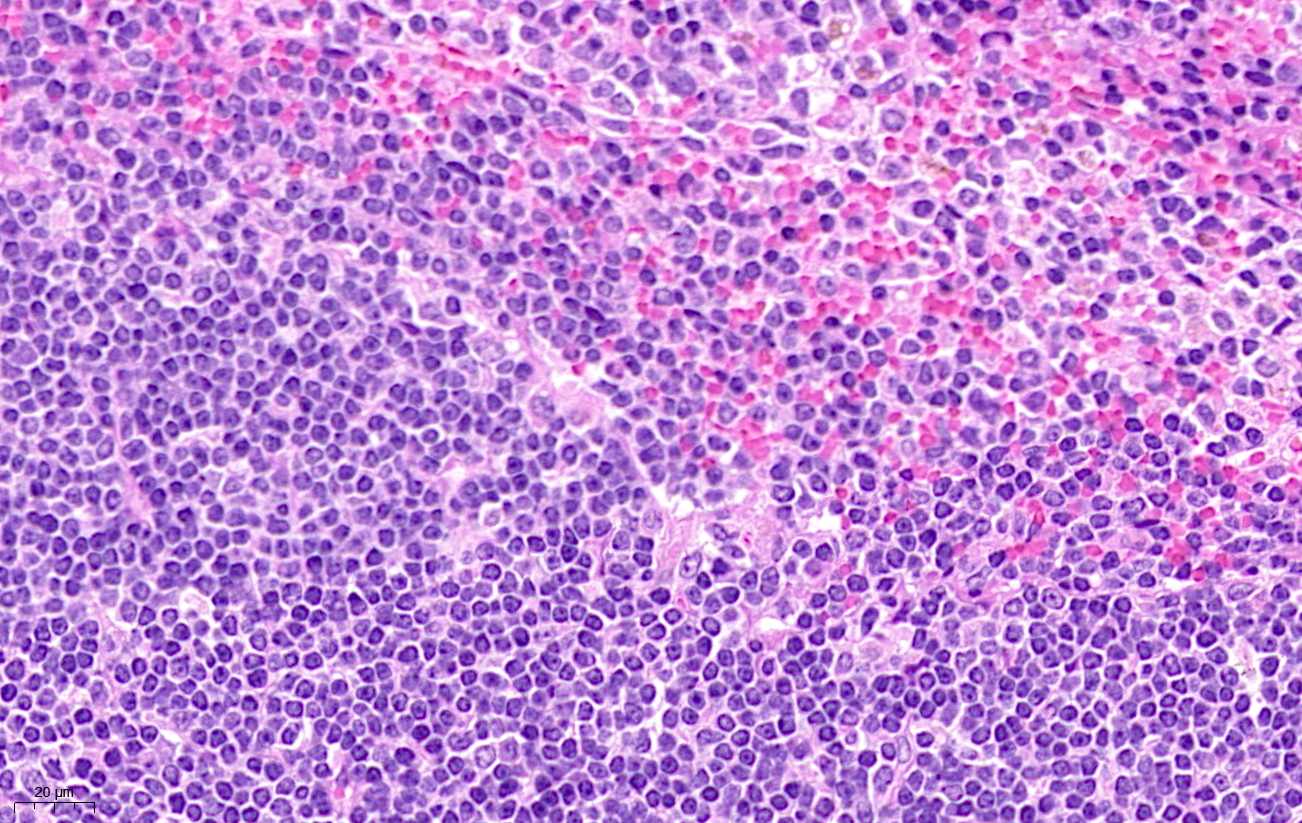

Supplement: Supplemental Material [file KBIE_A_2058149_SM0390.zip › supplementary/Untranstduced T cells group spleen HE staining.jpg]
